# Open Data Governance at the Canadian Open Neuroscience Platform (CONP): From the Walled Garden to the Arboretum

**DOI:** 10.1093/gigascience/giad114

**Published:** 2024-01-13

**Authors:** Alexander Bernier, Bartha M Knoppers, Patrick Bermudez, Michael J S Beauvais, Adrian Thorogood, CONP Consortium, Brendan Behan, Pierre Bellec, Shawn Brown, David Bujold, Ann Cavoukian, John Clarkson, Samir Das, Emilie Dessureault, Moyez Dharsee, Erin Dickie, Simon Duchesne, Stephanie Dyke, Ken Evans, Alan Evans, Jennifer Flynn, Nils Forkert, Tom Gee, Tristan Glatard, Richard Gold, Rachel Harding, Felipe Henriques, Sean Hill, Judy Illes, Jason Karamchandani, Ali Khan, Greg Kiar, Bartha Maria Knoppers, Xavier Lecours, Melanie Legault, Dave MacFarlane, Cécile Madjar, Roland Nadler, Santiago Paiva, Paul Pavlidis, Jean-Baptiste Poline, David Rotenberg, Marc-Etienne Rousseau, Walter Stewart, Nikola Stikov, Elizabeth Theriault, Alan Evans

**Affiliations:** Centre of Genomics and Policy, Department of Human Genetics, Faculty of Medicine and Health Sciences, McGill University, 740, Dr Penfield Ave, suite 5200, Montréal, Québec H3A 0G1, Canada; Centre of Genomics and Policy, Department of Human Genetics, Faculty of Medicine and Health Sciences, McGill University, 740, Dr Penfield Ave, suite 5200, Montréal, Québec H3A 0G1, Canada; McGill Centre for Integrative Neuroscience, Montreal Neurological Institute, McGill University, Montréal, Québec H3A 2B4, Canada; Faculty of Law, University of Toronto, Falconer Hall, 84 Queens Park, Toronto, Ontario M5S 2C5, Canada; The Terry Fox Research Institute, 110 Pine Ave W, Montreal, Quebec H2W IR7, Canada; McGill Centre for Integrative Neuroscience, Montreal Neurological Institute, McGill University, Montréal, Québec H3A 2B4, Canada

## Abstract

Scientific research communities pursue dual imperatives in implementing strategies to share their data. These communities attempt to maximize the accessibility of biomedical data for downstream research use, in furtherance of open science objectives. Simultaneously, such communities safeguard the interests of research participants through data stewardship measures and the integration of suitable risk disclosures to the informed consent process. The Canadian Open Neuroscience Platform (CONP) convened an Ethics and Governance Committee composed of experts in bioethics, neuroethics, and law to develop holistic policy tools, organizational approaches, and technological supports to align the open governance of data with ethical and legal norms. The CONP has adopted novel platform governance methods that favor full data openness, legitimated through the use of robust deidentification processes and informed consent practices. The experience of the CONP is articulated as a potential template for other open science efforts to further build upon. This experience highlights informed consent guidance, deidentification practices, ethicolegal metadata, platform-level norms, and commercialization and publication policies as the principal pillars of a practicable approach to the governance of open data. The governance approach adopted by the CONP stands as a viable model for the broader neuroscience and open science communities to adopt for sharing data in full open access.

## Introduction

Open science promotes the open dissemination of data, software, materials, manuscripts, and other outputs of scientific research to make them more transparent, accessible, and reproducible. A broad cross section of international bodies, including the OECD and UNESCO, have recognized the potential for open science to benefit both the general public and scientific research communities [1–3]. The justifications for open science practices are wide-ranging and multidimensional and remain subject to ongoing community debate and elaboration. Oft-cited considerations include enabling public participation in defining research questions and performing scientific research, reducing the barriers to accessing research materials, and ensuring scientific accountability.

The siloed storage of biomedical research data hinders the pursuit of accessible, inclusive, and reproducible research. Greater openness in the sharing of data enables community-wide collaboration to improve the reproducibility of findings, conduct large-scale agglomeration of data that enhance statistical power, and improve the representation of underserved populations [4]. The Canadian Open Neuroscience Platform (CONP) is among an increasing number of international initiatives working to develop policy standards for the open and unrestricted sharing of human biomedical research data. Other examples of organizations that have fostered the development of policies, standards, and tools that facilitate the interoperable sharing of neuroscience data include the Personal Genome Project, the *GigaScience* GigaDB, the Human Cell Atlas, the Global Alliance for Genomics and Health (GA4GH), and the International Neuroinformatics Coordinating Facility (INCF) [5–12]. The CONP has developed policies, practices, and technological tools for the open-access sharing of neuroscience data. Its approach rests on international bioethics norms, responds to regulatory requirements, and builds upon principles of open science, neuroethics, and privacy by design. The CONP emerged in Canada, but it proposes approaches to open data sharing that can nonetheless be translated to other jurisdictions and other data types.

In recognition of the value of sharing results and lessons learned with a growing community that is facing similar and substantive challenges in developing and implementing open data-sharing practices, this article details the open-data governance policies of the CONP and the tools that enable concordant practices. Part 1 describes the technical aspects of the CONP Portal (data- and tool-sharing infrastructure) and the premises underlying its submission policies. Part 2 states the CONP’s governance principles and the tools used to ensure that data submission is performed in a manner that respects established bioethics principles.

## Part 1: CONP Data- and Tool-Sharing Infrastructure and Its Governance

The CONP Portal is a purpose-built data- and tool-sharing platform that allows data to reside on different infrastructures through its flexible distributed management system. Portal users can choose among different methods of hosting their data, including third-party storage provided by the OSF, Zenodo, or storage native to the CONP technical infrastructure (via its “Community Server”), and can benefit from even greater flexibility in data hosting location through the combined use of the DataLad distributed data management system [13] and the GitHub open software repository to host the dataset metadata. (Persistence policies for repositories such as GitHub can change at any moment. For this and other reasons, such as facilitating provenance tracking, metadata are also stored locally on the CONP Portal’s servers and accompany every dataset, whether through browser-based or DataLad access.) This enables the CONP to host data residing on both its own technical infrastructure and external data repositories, with the distinction being transparent to the user who can browse, search for, and access data irrespective of storage site. These design choices also give data depositors greater flexibility by not obligating them to upload their data to a single, centralized point, which may not be possible because of technical or legal impediments. Further features provide both browser-based and command-line access to data, as well as a pathway to high-performance computing via the CBRAIN interface [14].

The CONP Portal’s technical design has implications for data stewardship. The CONP requires data contributors that host their data directly on CONP infrastructure to adhere to the stewardship standards that are detailed in its Consent Guide and its Privacy and De-Identification Guide [15]. Currently, CONP-hosted data are made available in full open access and are therefore available to all members of the public.

For data that are findable and accessible through the CONP Portal but not stored on its Community Server, it is sufficient for contributors to respect their local legal and biomedical research ethics requirements and the data stewardship policies of the selected host repository. In this latter case, adherence to the CONP data stewardship guidance is recommended but not required as, from a data governance standpoint, it is the stewardship practices of the host repository that ensure that externally hosted data are subject to appropriate oversight, and it is unnecessary for the CONP to also mandate compliance with its own data stewardship practices. Conversely, the CONP requires adherence to its data governance standards for data it hosts natively and for which it therefore takes on the role of primary data steward.

This combination of technical and policy design enables the CONP to stipulate hosting conditions for all data residing on its own infrastructure while enabling external repositories to make their data discoverable and downloadable through the CONP, despite such data being held according to distinct data governance standards, including in registered or controlled access. This ensures that data that are useful for common research purposes can still be found and accessed through a singular data portal with the benefit of harmonized metadata.

**Figure 1:**
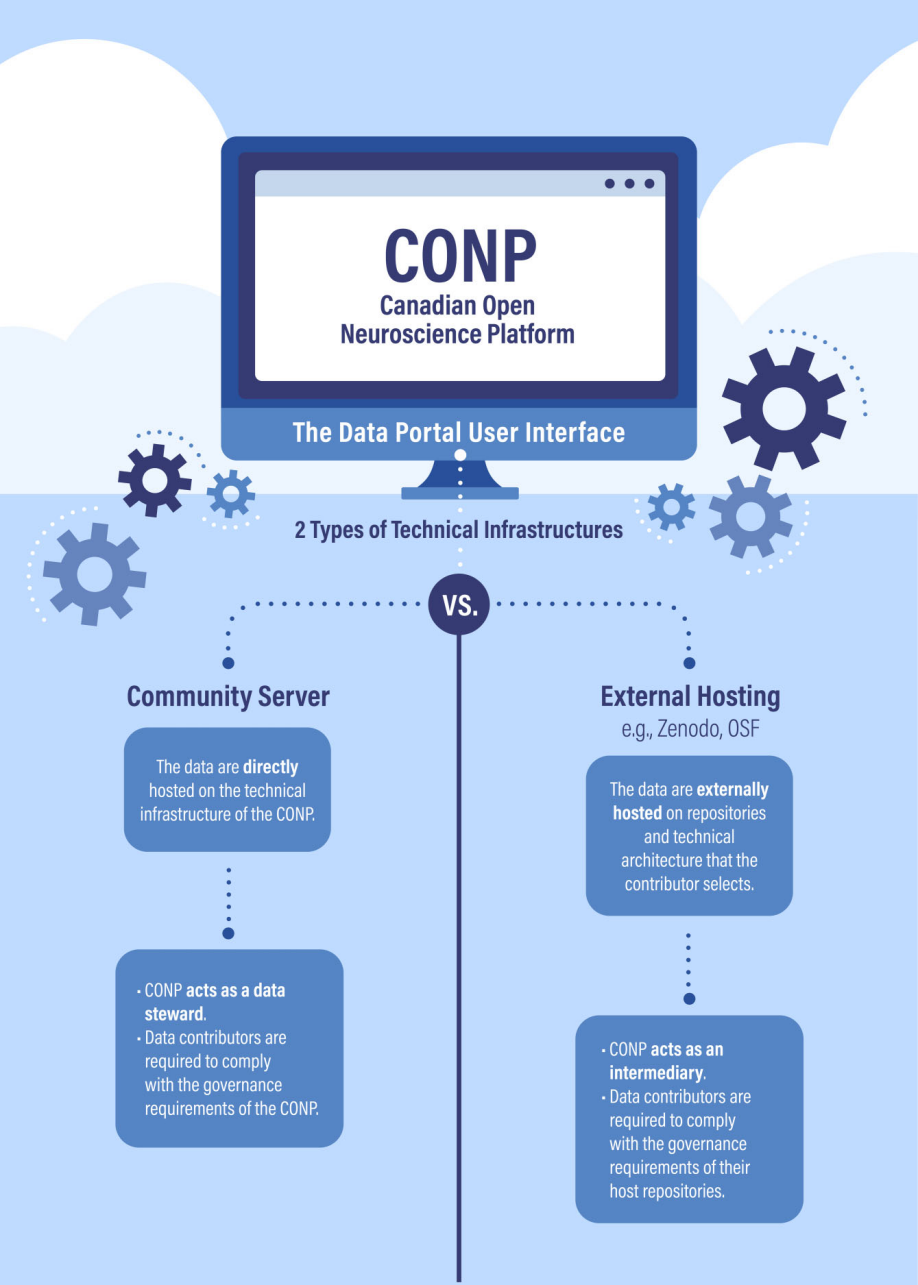
CONP Data Portal Technical Infrastructures (comparison).

In sum, the CONP’s technical infrastructure allows it to span functionality that ranges between a traditional centralized data repository and a decentralized discovery tool that operates across multiple distinct repositories. The stewardship practices of the CONP align with and support its choice of technical architecture, enable centralized hosting and access to similarly permissioned data, and allow discovery and download of externally hosted and distinctly permissioned data (see Fig. 1). This achieves a compromise between competing policy imperatives: incentivizing data contributors to adopt similar data conditions of data governance and enabling data that are not subject to harmonized data governance conditions to be discovered through a single platform [16].

## Part 2: Conceptualizing Data Governance Practices for Open-Access Data Sharing

The shift from holding coded human biomedical data in controlled access to full open access requires a correlative change in the governance measures that are used to safeguard the rights and interests of individuals who consent to have their data hosted in the concerned repositories. Declining costs in information processing, data storage, and data analysis have made viable large-scale, data-intensive biomedical research that leverages existing data from multiple repositories. This has produced a concomitant shift in the practical application of international biomedical research ethics principles. In the past, informed consent materials and data governance practices restricted the use of research data to the research project in which they were generated and narrowly limited reuse to other closely related research efforts [17]. This reflected the high costs of and limited technical prospect for repurposing data outside the research project for which it was originally generated. As the cost-effective aggregation of large quantities of biomedical research data became technically possible, tools and career specializations to support data interoperability proliferated (e.g., dedicated personnel trained to perform data harmonization and data management, stakeholder forums dedicated to elaborating and refining shared ontologies, file formats, and other interoperability standards) [18–20]. Corresponding changes in biomedical research ethics practice and data-sharing policies followed.

International biomedical research ethics instruments, such as the World Medical Association’s Declaration of Taipei, increasingly legitimate the indefinite storage of biomedical research data in databases and repositories for long-term reuse. Research communities have started to shift from obtaining purpose-limited and time-limited consent to securing broad consent to the ongoing use of data, conditional on its continued stewardship [21]. This governance approach is often realized through the use of a controlled-access mechanism, through which researchers obtain informed consent or other authorizations to remove the most conspicuous individual identifiers from the data to mitigate privacy risks (i.e., the data are coded) and subsequently deposit the deidentified data in repositories for their long-term retention and future use. Data stewards bring together relevant bioethics and legal expertise, scientific knowledge of the concerned field of research, and technical skills relevant to the operation and management of the host database. These actors perform oversight and devise general policies that determine how that data can be used. Accredited researchers can then submit applications to these governance bodies for access to data for a specified period of time and for a specified purpose.

Controlled access mechanisms leverage data deidentification and ongoing control of downstream data use to minimize the residual risks of privacy infringement and information misuse. The administrative burdens associated with the submission and oversight of data access requests, however, can prevent the scalable use of data across multiple biorepositories, as well as greatly reducing the likelihood of wider and deeper exploration of valuable existing data by the scientific community. Furthermore, because instituting and maintaining access committees is labor- and resource-intensive, the choice to hold data according to controlled access can create challenges for the long-term financial and operational sustenance of such repositories [22].

Navigating controlled access processes creates considerable administrative burdens for researchers, especially those in low-and middle-income countries or outside traditional academic research organizations (e.g., small and medium enterprises (SMEs) or citizen scientists) [23, 24]. In decreasing the transaction costs inherent to accessing data, open access makes it possible for a broader community of researchers to perform studies with greater statistical power. Relative to controlled access, the deposit of data in full open access also aligns with the wishes of many research participants and communities to do so, maximizing their contribution to science. Open access may also hold the potential to reduce disparities in access to the benefits of research to the degree that it maximizes accessibility to data from underrepresented subpopulations.

Though the sharing of data subject to controlled access mechanisms and other restrictions on data use will remain a standard data stewardship practice for the foreseeable future, demand on the part of researchers and research participants, as well as the increasing benefits of data use at scale, favors the creation of data repositories dedicated to sharing biomedical research data in full open access as a default practice. The greatest challenge thereto is to create data stewardship processes that are suitable to data that will be held for long-term future use in open, public repositories, for which no oversight of the case-specific uses made of open-access data can be performed.

The CONP enables full open access to research data by leveraging participants’ informed consent and the use of data deidentification requirements to further mitigate the risk of individual reidentification or harmful data use. This shifts the core of the governance approach from postingestion active stewardship to rigorous preingestion informed consent and data deidentification. This governance model responds to prevailing legal paradigms and applicable ethics requirements to enable the open sharing of neuroscience data that are consented or otherwise permissioned for open release. The mitigation of population-specific or group-specific harms is mediated through the involvement of research ethics boards (REBs) that oversee research or through other stakeholders, such as patient communities and population-specific research organizations, prior to the upload of individuals’ deidentified data to the CONP. These actors mitigate such risks in overseeing the drafting of informed consent materials, determining how data can be collected, deidentified, and released, and assessing whether a particular data repository is suitable for the deposit of data.

In the future, collaboration with stakeholders from relevant populations will be required for the CONP to tailor its governance approach to concerns that are specific to certain vulnerable groups or communities of patients with unique needs. For example, the CONP can seek to engage with indigenous communities to explore the degree to which open-access approaches might align with community interests and data sovereignty. Population-specific governance modifications could include adjusting data governance and consent procedures to account for population-specific concerns, for example, to enable data contribution from those who do not have legal capacity to provide informed consent on their own behalf (e.g., pediatric populations or patients with neurodegenerative diseases), or implementing population-specific deidentification or data manipulation processes that mitigate relevant group-level harms. This will further enable the dissemination of data relative to populations underrepresented in research datasets through the CONP Portal.

### The CONP Ethics and Data Governance Committee

The CONP Ethics and Governance Committee, composed of experts in bioethics, neuroethics, and law, has produced a CONP Governance Framework [25] that establishes the central concepts and principles that inform CONP governance policies, as well as guidance to translate these principles into immediate practice and long-term objectives for the governance of open data that require additional deliberation to implement. The Framework’s guiding principles are (i) researcher integrity, (ii) autonomy, (iii) privacy, (iv) scope of data access and use, (v) capacity to consent, (vi) participant health, (vii) community engagement, and (viii) trust and accountability. The Governance Framework incorporates detailed subpoints articulating each principle and translating them into applicable rules or expanding upon the values that each reflects.

Drawing from the Governance Framework, the CONP has developed consent and privacy and deidentification guides detailing ethics and data governance requirements that apply to prospective data contributors. Together, these latter 2 documents form the CONP Ethics Toolkit. As discussed above, respect thereof is required for data contributors that store their data on CONP servers. The CONP has further innovated in creating metadata elements that can be associated to datasets to describe the conditions of use associated thereto in a manner that will trail the data as they are downloaded from the CONP. Below, we describe these elements in detail. In addition to performing the foregoing functions, the Ethics and Data Governance Committee provides *ad hoc* guidance to the operational staff of the CONP and to researchers who intend to deposit data on the CONP Portal, responding to governance challenges as these arise. This includes tailoring the CONP guidance tools to respond to new risks or requirements and providing counsel on their application. The Ethics and Data Governance Committee further monitors for potential risks associated to the upload of specific categories of data, specific populations, or circumstances of data upload that might require the imposition of additional governance controls on a contextual basis, especially where communities indicate that data pertaining to their members require additional governance safeguards to mitigate the potential for public data dissemination to lead to group-level harms [15, 26, 27].

### The CONP Consent Guide

Obtaining informed consent is a precursor to performing scientific research involving human participants and their identifiable data. The information provided to participants during the informed consent process often determines the conditions according to which acquired data can be used for future research purposes. Reusing data in a manner outside the scope of an existing informed consent often requires considerable investment in either obtaining confirmation from an REB that it is ethical to proceed absent a new consent or seeking a new informed consent from the concerned individuals.

The CONP Consent Guide provides guidance for researchers obtaining informed consent to the collection of data for the purpose of submitting it to the CONP Portal’s Community Server or in determining whether an existing informed consent is suitable for such a submission. It has 3 main components. First, it contains a list of core consent elements that must be reflected in the informed consent materials of research studies that intend to contribute data to the CONP Portal’s Community Server. Second, the guide contains a retrospective consent filter. This is a self-assessment tool that enables researchers to determine whether they have included the necessary elements for open data sharing in their study’s informed consent form (ICF) and their data can be contributed to the Community Server as-is, or if additional steps might be required before such data are suitable for contribution to the CONP. Other biomedical research consortia, such as the Human Cell Atlas (HCA) and the International Cancer Genome Consortium (ICGC), have used retrospective consent filters to guide researchers in depositing data in open access [28]. Third, the CONP Consent Guide also contains template clauses that reflect the foregoing core consent elements, which researchers can adapt to meet local regulatory requirements or institutional demands.

The CONP Core Consent Elements are as follows:

1. Generation of participant data for research purposes
2. Data deidentification (i.e., coding, anonymization, or synthetic data generation)
3. Sharing of deidentified data via the CONP Portal, an open-access platform that researchers the world over may access
4. Deidentified data that can be used for commercial purposes
5. Not possible to withdraw data that have already been shared
6. Low risk that the participant could be reidentified in the future

These core consent elements contain broad permissions that allow data to be stored in open platforms that scientists and the general public can use for research purposes without imposing major limitations on how data may be used. Further, the information provided enables research participants to understand the risks inherent to their data being used and to appreciate the limits on a potential withdrawal of the submitted data. These elements are derived from the generalist clauses of the GA4GH and, therefore, can be used in a manner that is interoperable with other data that have been collected according to GA4GH standards or close derivatives thereof [29]. This approach builds on the implementation of broad consent to data sharing in other large-scale biomedical research consortia, leveraging appropriate risk disclosure, consent to broad data sharing, and data deidentification to disclose and mitigate the potential privacy risks associated with data sharing [21]. The governance strategy of the CONP consists in using risk disclosures and data deidentification to communicate and mitigate risks of individual reidentification, rather than performing ongoing governance of data access requests. The foregoing consent guidance therefore requires researchers to inform the research participants that their data will be shared with the public to enable open research and that there remains a small residual risk that their data could be reidentified in the future. In contrast to the pairing of broad consent and use-specific access controls, the CONP’s approach to data governance emphasizes risk communication and data deidentification as its principal data stewardship mechanisms.

### The CONP Privacy and Deidentification Guide

The deidentification of data can often be an ethical or legal precondition to its continued use or its transmission to third parties. Deidentification is a context-specific procedure that requires data contributors or data stewards to remove or transform (e.g., generalize) the features of a dataset that could enable individual reidentification and those that are highly sensitive and potentially detrimental to the individual. The CONP has developed a Privacy and Deidentification Guide that helps researchers establish how data should be deidentified prior to their submission to the CONP Portal’s Community Server. It requires data contributors to reduce the risk of individual reidentification to a low residual likelihood prior to submitting data to the CONP for public disclosure. This guide is also intended to propose standard mechanisms for assessing data identifiability and for performing data deidentification that other open neuroscience communities can adopt. It restates key concepts from Canadian research ethics guidance, concepts from the regulatory guidance of Canadian privacy commissioners, and concepts established in data protection law.

To help scientists reduce the risk of individual reidentification as much as possible while maintaining the scientific utility of the data, the CONP Privacy and Deidentification Guide provides links to resources that are tailored to neuroscience data, including deidentification guidance or algorithms that remove identifying information, such as names or birthdates from data-file headers or facial features from magnetic resonance images. It also recommends tools that enable the generation of synthetic data and help researchers assess whether their data are best held in controlled access, registered access, or open-access repositories according to their sensitivity and associated risk of reidentification [30].

### Ethics provenance metadata and the data upload process

Platforms that host data for secondary use are required to communicate to data contributors their responsibility to obtain required authorizations prior to depositing data and for compliance with the platform’s data submission policies. More onerous methods of managing data submission include the use of contracts and data contribution forms that are subject to expert review prior to the upload of data to a platform, sometimes requiring attestations and signatures from authorized representatives of the submitting institution. Less onerous mechanisms include the use of “click-wrap” agreements that require data contributors to assert their understanding of and compliance with the preconditions of data submission, which pop up on the screen of the contributor as part of the data submission process [31].

The data upload form requires (i) an attestation that 1 of 4 acceptable conditions for data upload has been satisfied (including participants have provided a valid informed consent to the deidentification and deposit of their data in an open-access portal, a waiver or other authorization to deposit these deidentified data in an open-access portal was obtained from a research ethics body [research ethics board (REB), institutional review board (IRB), research ethics committee (REC), etc.], local law or a relevant institutional authorization otherwise enables the deposit of these data in an open-access portal, or these data are not derived from human participants), (ii) the parties uploading the data to specify which open intellectual property license has been applied to their data, and (iii) uploaders to stipulate whether the data are held in open access, registered access, or controlled access. For data hosted directly on the CONP’s technical infrastructure, open access is currently the only option. Last, for those datasets that attest that an REB has performed the oversight of their data, the applicable REB approval number is also provided as a measure of evidence that their data have genuinely been subject to an REB evaluation.

The CONP Portal data submission procedure requires data contributors to provide a minimal set of metadata (implemented in the form of the standard Data Tags Suite model) [32] along with their dataset. A subset of these metadata collects information about the conditions of use applicable to the data and ensure that the preconditions to hosting data on the CONP Portal are satisfied, thereby prompting contributors to hold themselves accountable for their use of data.

## Conclusion

The CONP’s data governance policies and tools emphasize presubmission informed consent practices, robust data deidentification tools, and the inclusion of ethicolegal metadata with shared data. The CONP therefore enables researchers to submit datasets in full open access in compliance with their ethical, legal, and institutional commitments. This allows for increased pluralism in approaches to data stewardship represented among biomedical data repositories. Its approach provides a greater range of options to research participants and researchers in selecting the combination of data access controls, deidentification practices, and community rules that best align with their preferences and the ethical and legal commitments of their local institution. It is hoped that the CONP’s approach to data stewardship might also serve as a model for other open neuroscience initiatives in Canada and elsewhere.

## Supplementary Material

giad114_GIGA-D-23-00204_Original_Submission

giad114_GIGA-D-23-00204_Revision_1

giad114_GIGA-D-23-00204_Revision_2

giad114_Response_to_Reviewer_Comments_Original_Submission

giad114_Response_to_Reviewer_Comments_Revision_1

giad114_Reviewer_1_Report_Original_Submission Anita Bandrowski -- 8/11/2023 Reviewed

giad114_Reviewer_1_Report_Revision_1 Anita Bandrowski -- 11/26/2023 Reviewed

giad114_Reviewer_2_Report_Original_Submission Jie Yin, Ph.D -- 8/15/2023 Reviewed

giad114_Reviewer_3_Report_Original_Submission Angela Jean Ballantyne -- 8/20/2023 Reviewed

## Data Availability

No data was generated or used in the drafting of this article and therefore this consideration is not applicable to the manuscript.

## Abbreviations

CONP: Canadian Open Neuroscience Platform; GA4GH: Global Alliance for Genomics and Health; HCA: Human Cell Atlas; ICF: informed consent form; ICGC: International Cancer Genome Consortium; INCF: International Neuroinformatics Coordinating Facility; ROB: research ethics board.

## Notes

CONP Consortium (listed alphabetically): Brendan Behan, Pierre Bellec, Shawn Brown, David Bujold, Ann Cavoukian, John Clarkson, Samir Das, Emilie Dessureault, Moyez Dharsee, Erin Dickie, Simon Duchesne, Stephanie Dyke, Ken Evans, Alan Evans, Jennifer Flynn, Nils Forkert, Tom Gee, Tristan Glatard, Richard Gold, Rachel Harding, Felipe Henriques, Sean Hill, Judy Illes, Jason Karamchandani, Ali Khan, Greg Kiar, Bartha Maria Knoppers, Xavier Lecours, Melanie Legault, Dave MacFarlane, Cécile Madjar, Roland Nadler, Santiago Paiva, Paul Pavlidis, Jean-Baptiste Poline, David Rotenberg, Marc-Etienne Rousseau, Walter Stewart, Nikola Stikov, and Elizabeth Theriault.

## References

[1] Organisation for Economic Cooperation and Development (OECD) . Open Science: Enabling Data Discovery in the Digital Age. Going Digital Toolkit Note, No. 13, Paris: OECD Publishing 2021. 10.1787/81a9dcf0-en

[2] Organisation for Economic Cooperation and Development (OECD) . Recommendation of the Council Concerning Access to Research Data from Public Funding. 2021. https://www.oecd.org/sti/recommendation-access-to-research-data-from-public-funding.htm

[3] United Nations Economic, Social, and Cultural Organisation (UNESCO) . UNESCO Recommendation on Open Science. ​​​​​​ Paris: UNESCO 2021. 10.54677/MNMH8546

[4] Gilmore RO , Xu M, Adolph KE. Data sharing. In: Panicker S, Stanley B, eds. Handbook of Research Ethics in Psychological Science. Washington D.C.: American Psychological Association, 2021:83–97. ISBN: 978-1-4338-3636-7

[5] Regev A , Teichmann S, Rozenblatt-Rosen O, et al. The Human Cell Atlas White Paper. Human Cell Atlas. Online. Available from: https://arxiv.org/abs/1810.05192. 2018. 10.48550/arXiv.1810.05192.

[6] Ball MP , Bobe JR, Chou MF, et al. Harvard Personal Genome Project: lessons from participatory public research. Genome Med. 2014;6:10. 10.1186/gm527. 24713084 PMC3978420

[7] Sneddon TP , Li P, Edmunds SC. GigaDB: announcing the GigaScience database. Gigascience. 2012;1:1. 10.1186/2047-217X-1-11. 23587345 PMC3626507

[8] Abrams MB , Bjaalie JG, Das S, et al. A standards organization for open and FAIR neuroscience: the international neuroinformatics coordinating facility. Neuroinform. 2022;20(1):25–36. 10.1007/s12021-020-09509-0. PMC9036053 33506383

[9] Martone ME . A decade of GigaScience: the importance of community organizations for open and FAIR efforts in neuroinformatics. Gigascience. 2022;11:giac060. 10.1093/gigascience/giac060. 35701370 PMC9197677

[10] Rahimzadeh V , Dyke SO, Knoppers BM. An international framework for data sharing: moving forward with the global alliance for genomics and health. Biopreserv Biobanking. 2016;14(3):256–9. 10.1089/bio.2016.0005. 27082668

[11] Knoppers BM . International ethics harmonization and the global alliance for genomics and health. Genome Med. 2014;6(2):1–3. 10.1186/gm530. 25031613 PMC3979077

[12] Valdes-Sosa PA , Leon MAB, Lopera F, et al. EEG in the Global Brain Consortium, aiming to strengthen linkages between neuroscientists across borders and disciplines to advance equitable solutions to priority health challenges worldwide. Alzheimers Dementia. 2022;18:e059945. 10.1002/alz.059945

[13] Halchenko Y , Meyer K, Poldrack B, et al. DataLad: distributed System for Joint management of code, data, and their relationship. JOSS. 2021;6(63):3262. 10.21105/joss.03262. PMC11514317 39469147

[14] Sharif T , Rioux P, Rousseau M, et al. CBRAIN: a web-based, distributed computing platform for collaborative neuroimaging research. Front Neuroinform. 2014;8:54. 10.3389/fninf.2014.00054 24904400 PMC4033081

[15] Beauvais MJS , Illes J, Knoppers BM. A marathon, not a sprint—neuroimaging, open science and ethics. Neuroimage. 2021;236:118041. 10.1016/j.neuroimage.2021.118041. 33848622

[16] Thorogood A . Policy-aware Data lakes: a flexible approach to achieve legal interoperability for global research collaborations. J Law Biosci. 2020;7:1. 10.1093/jlb/lsaa065. PMC7454728 33005429

[17] Bernier A , Knoppers BM. Longitudinal Health studies: secondary uses serving the future. Biopreserv Biobanking. 2021;19(5):404–13. 10.1089/bio.2020.0171. 34171963

[18] Luo J , Wu M, Gopukumar D, et al. Big data application in biomedical research and health care: a literature review. Biomed Inform Insights. 2016;8:BII–S31559. 10.4137/BII.S31559. PMC4720168 26843812

[19] Navale V , Bourne PE. Cloud computing applications for biomedical science: a perspective. PLoS Comput Biol. 208;14(6):e1006144. 10.1371/journal.pcbi.1006144. 29902176 PMC6002019

[20] Cirillo D , Valencia A. Big data analytics for personalized medicine. Curr Opin Biotechnol. 2019;58:161–7. 10.1016/j.copbio.2019.03.004. 30965188

[21] Boers SN , van Delden JJN, Bredenoord AL. Broad consent is consent for governance. Am J Bioeth. 2015;15(9):53–55. 10.1080/15265161.2015.1062165. 26305756

[22] Devriendt T , Borry P, Shabani M. Factors that influence data sharing through data sharing platforms: a qualitative study on the views and experiences of cohort holders and platform developers. PLoS One. 2021;16(7):e0254202. 10.1371/journal.pone.0254202. 34214146 PMC8253381

[23] Corpas M , Kovalevskaya NV, McMurray A, et al. A FAIR guide for data providers to maximise sharing of human genomic data. PLoS Comput Biol. 2018;14(3):e1005873. 10.1371/journal.pcbi.1005873. 29543799 PMC5854239

[24] Shabani M , Borry P. “You want the right amount of oversight”: interviews with data access committee members and experts on genomic data access. Genet Med. 2016;18:892–7. 10.1038/gim.2015.189. 26795589

[25] Cavoukian A , Clarkson J, Flynn J, Gold R, Illes J, Knoppers BM, Nadler R, Stewart W, Thorogood A. CONP Ethics and Data Governance Framework. Canadian Open Neuroscience Platform; 2019. https://conp.ca/ethics-governance/.

[26] Mittelstadt B . From individual to group privacy in big data analytics. Philosophy Technol. 2017;30(4):475–94.. 10.1007/s13347-017-0253-7.

[27] Bloustein EJ . Group privacy: the right to huddle. Rutgers-Cam LJ. 1976;8:219.

[28] Wallace SE , Kirby E, Knoppers BM. How can we not waste legacy genomic data?. Front Genet. 2020;11:446. 10.3389/fgene.2020.00446. 32457803 PMC7225345

[29] Global Alliance for Genomics and Health . Consent Clauses for Genomic Research. Global Alliance for Genomics and Health Regulatory and Ethics Toolkit. 2020. https://www.ga4gh.org/document/consent-clauses-for-genomic-research/.

[30] Knoppers BM , Beauvais MJS, Cavoukian A, et al. Canadian Open Neuroscience Platform (CONP) Ethics Toolkit. 2022. https://conp.ca/ethics-toolkit/.

[31] Bernier A , Molnár-Gábor F, Knoppers BM. The international data governance landscape. J Law Biosci. 2022;9(1):lsac005. 10.1093/jlb/lsac005. 35382430 PMC8977111

[32] Sansone SA , Gonzalez-Beltran A, Rocca-Serra P, et al. DATS, the data tag suite to enable discoverability of datasets. Sci Data. 2017;4:170059. 10.1038/sdata.2017.59. 28585923 PMC5460592

