# Supplementary material for: Open Data Governance at the Canadian Open Neuroscience Platform (CONP): From the Walled Garden to the Arboretum
Source: Gigascience. 2024 Jan 13;13:giad114. doi: 10.1093/gigascience/giad114 (PMC10787360; doi:10.1093/gigascience/giad114)
Supplement: giad114_GIGA-D-23-00204_Original_Submission [file giad114_giga-d-23-00204_original_submission.pdf]

GigaScience

Open Data Governance at the Canadian Open Neuroscience Platform (CONP): From the Walled Garden to the Arboretum

--Manuscript Draft--

Manuscript Number:	GIGA-D-23-00204
Full Title:	Open Data Governance at the Canadian Open Neuroscience Platform (CONP): From the Walled Garden to the Arboretum
Article Type:	Review
Funding Information:	Fondation Brain Canada — Prof Alan Evans
Abstract:	Scientific research communities pursue dual imperatives in implementing strategies to share their data. These communities attempt to maximize the accessibility of biomedical data for downstream research use, in furtherance of open science objectives. Simultaneously, such communities safeguard the interests of research participants through data stewardship measures and the integration of suitable risk disclosures to the informed consent process. The Canadian Open Neuroscience Platform (CONP) convened an Ethics and Governance Committee composed of experts in bioethics, neuroethics, and law, to develop holistic policy tools, organisational approaches, and technological supports to align the open governance of data to ethical and legal norms. The CONP has adopted novel platform governance methods that favor full data openness, legitimated through the use of robust de-identification processes and informed consent practices. The experience of the CONP is articulated as a potential template for other open science efforts to further build upon. This experience highlights informed consent guidance, de-identification practices, ethico-legal metadata, platform-level norms, and commercialization and publication policies as the principal pillars of a practicable approach to the governance of open data. The governance approach which the CONP has adopted for its open data stands as a viable model for the broader open science community, and broader neuroscience community, to adopt in sharing data in full open access.
Corresponding Author:	Alexander Bernier, J.D. LL.M.<br>University of Toronto Faculty of Law<br>Toronto, CANADA
Corresponding Author Secondary Information:	
Corresponding Author's Institution:	University of Toronto Faculty of Law
Corresponding Author's Secondary Institution:	
First Author:	Alexander Bernier, J.D. LL.M.
First Author Secondary Information:	
Order of Authors:	Alexander Bernier, J.D. LL.M.<br>Bartha M. Knoppers, PhD<br>Patrick Bermudez, PhD<br>Michael J. S. Beauvais, BCL JD<br>Adrian Thorogood, BCL LLB LLM<br>CONP Consortium†<br>Alan Evans, PhD
Order of Authors Secondary Information:	
Additional Information:	
Question	Response

Powered by Editorial Manager® and ProduXion Manager® from Aries Systems Corporation

Are you submitting this manuscript to a special series or article collection?	No
**Experimental design and statistics**<br><br>Full details of the experimental design and statistical methods used should be given in the Methods section, as detailed in our Minimum Standards Reporting Checklist. Information essential to interpreting the data presented should be made available in the figure legends.<br><br>Have you included all the information requested in your manuscript?	Yes
**Resources**<br><br>A description of all resources used, including antibodies, cell lines, animals and software tools, with enough information to allow them to be uniquely identified, should be included in the Methods section. Authors are strongly encouraged to cite Research Resource Identifiers (RRIDs) for antibodies, model organisms and tools, where possible.<br><br>Have you included the information requested as detailed in our Minimum Standards Reporting Checklist?	Yes
**Availability of data and materials**<br><br>All datasets and code on which the conclusions of the paper rely must be either included in your submission or deposited in publicly available repositories (where available and ethically appropriate), referencing such data using a unique identifier in the references and in the "Availability of Data and Materials" section of your manuscript.<br><br>Have you have met the above requirement as detailed in our Minimum	Yes

Standards Reporting Checklist?	

Powered by Editorial Manager® and ProduXion Manager® from Aries Systems Corporation

Manuscript
Click here to access/download;Manuscript;Gigascience Manuscript II.docx

Open Data Governance at the Canadian Open Neuroscience Platform (CONP): From the Walled Garden to the Arboretum

Alexander Bernier[1], Bartha M. Knoppers,[1] Patrick Bermudez[2], Michael J. S. Beauvais[3], Adrian Thorogood [4], CONP Consortium[†], Alan Evans[2].

[1] Centre of Genomics and Policy, Department of Human Genetics, Faculty of Medicine and Health Sciences, McGill University, Montréal, Québec, Canada

[2] McGill Centre for Integrative Neuroscience, Montreal Neurological Institute, McGill University, Montréal, Québec, Canada

[3] Faculty of Law, University of Toronto, Toronto, Ontario, Canada

[4] The Terry Fox Research Institute, Montreal, Quebec, Canada

[†] CONP Consortium (listed alphabetically): Brendan Behan, Pierre Bellec, Shawn Brown, David Bujold, Ann Cavoukian, John Clarkson, Samir Das, Emilie Dessureault, Moyez Dharsee, Erin Dickie, Simon Duchesne, Stephanie Dyke, Ken Evans, Alan Evans, Jennifer Flynn, Nils Forkert, Tom Gee, Tristan Glatard, Richard Gold, Rachel Harding, Felipe Henriques, Sean Hill, Judy Illes, Jason Karamchandani, Ali Khan, Greg Kiar, Bartha Maria Knoppers, Xavier Lecours, Melanie Legault, Dave MacFarlane, Cécile Madjar, Roland Nadler, Santiago Paiva, Paul Pavlidis, Jean-Baptiste Poline, David Rotenberg, Marc-Etienne Rousseau, Walter Stewart, Nikola Stikov, Elizabeth Theriault.

Abstract: Scientific research communities pursue dual imperatives in implementing strategies to share their data. These communities attempt to maximize the accessibility of biomedical data for downstream research use, in furtherance of open science objectives. Simultaneously, such communities safeguard the interests of research participants through data stewardship measures and the integration of suitable risk disclosures to the informed consent process. The Canadian Open Neuroscience Platform (CONP) convened an Ethics and Governance Committee composed of experts in bioethics, neuroethics, and law, to develop holistic policy tools, organisational approaches, and technological supports to align the open governance of data to ethical and legal norms. The CONP has adopted novel platform governance methods that favor full data openness, legitimated through the use of robust de-identification processes and informed consent practices. The experience of the CONP is articulated as a potential template for other open science efforts to further build upon. This experience highlights informed consent guidance, de-identification practices, ethico-legal metadata, platform-level norms, and commercialization and publication policies as the principal pillars of a practicable approach to the governance of open data. The governance approach which the CONP has adopted for its open data stands as a viable model for the broader open science community, and broader neuroscience community, to adopt in sharing data in full open access.

Introduction

Open Science promotes the open dissemination of data, software, materials, manuscripts, and other outputs of scientific research to make them more transparent, accessible, and reproducible. A broad cross-section of international bodies, including the OECD and UNESCO, have recognized the prospect for open science to benefit both the general public and scientific research communities [1; 2; 3]. The justifications for open science practices are various and remain subject to ongoing community debate. Oft-cited considerations include enabling public participation in defining research questions and performing scientific research, reducing the barriers to accessing research materials, and ensuring scientific accountability.

The siloed storage of biomedical research data hinders the pursuit of accessible, inclusive, and reproducible research. Greater openness in the sharing of data enables community-wide collaboration to improve the reproducibility of important findings, conduct large-scale agglomeration of data that enhance statistical power, and improve the representation of underserved populations [4]. The Canadian Open Neuroscience Platform (CONP) is among an increasing number of international initiatives working to develop policy standards for the open and unrestricted sharing of human biomedical research data. Other examples include the Personal Genome Project, the GigaScience GigaDB, and the Human Cell Atlas [5; 6; 7].

The CONP has developed policies, practices, and technological tools for the open-access sharing of neuroscience data. Its approach rests on international bioethics norms, responds to regulatory requirements, and builds upon principles of open science, neuroethics, and privacy by design. The CONP emerged in Canada and responds to the technical particularities of neuroscience data sharing. Nonetheless, it is intended to be a generalist approach to open data sharing that can be translated to other jurisdictions and other data types.

In recognition of the value of sharing results and lessons learned with a growing community that is facing similar and substantive challenges in developing and implementing open data-sharing practices, this article details the open-data governance policies of the CONP and the tools that enable concordant practices. Part One describes the technical aspects of the CONP Portal (data- and tool-sharing infrastructure) and the premises underlying its submission policies. Part Two states the CONP's governance principles and the tools used to ensure that data submission is performed in a manner that respects established bioethics principles.

Part One: CONP data- and tool-sharing infrastructure and its governance

The CONP Portal is a purpose-built data- and tool-sharing platform that allows data to reside on different infrastructures through its flexible distributed management system. Portal users can choose among

different methods of hosting their data, including third-party storage provided by the OSF, Zenodo, or storage native to the CONP technical infrastructure (via its 'Community Server') and can benefit from even greater flexibility in data hosting location through the combined use of the DataLad distributed data-management system [8] and the GitHub open software repository to host the dataset metadata. This enables the CONP to host data residing on both its own technical infrastructure and external data repositories, with the distinction being transparent to the user who can browse, search for, and access data irrespective of storage site. These design choices also give data depositors greater flexibility by not obligating them to upload their data to a single, centralized point, which may not be possible because of technical or legal impediments. Further features also provide both graphical, browser-based and command-line access to data, as well as a pathway to high-performance computing via the CBRAIN interface [9].

The CONP Portal's technical design has implications for data stewardship. The CONP requires data contributors that host their data directly on CONP infrastructure to adhere to the stewardship standards that are detailed in its Consent Guide and its Privacy and De-Identification Guide [10]. Currently, CONP-hosted data are made available in full open access and are therefore available to all members of the public.

For data that are findable and accessible through the Portal but stored on non-CONP servers, it is sufficient for contributors to respect their local legal and biomedical research ethics requirements and the data stewardship regulations of the selected host repository. In this latter case, adherence to the CONP data stewardship guidance is recommended but not required as, from a data governance standpoint, it is the stewardship practices of the host repository that ensure that externally hosted data are subject to appropriate oversight, and it is unnecessary for the CONP to also mandate compliance with its own data stewardship practices. Conversely, the CONP requires adherence to its data governance standards for data it hosts natively and for which it therefore takes on the role of primary data steward.

This combination of technical and policy design enables the CONP to stipulate hosting conditions for all data residing on its own infrastructure while enabling external repositories to make their data discoverable

and downloadable through the CONP, despite such data being held according to distinct data governance standards, including in registered or controlled access. This ensures that data that are useful for common research purposes can still be found and accessed through a singular data portal with the help of harmonized metadata.

In sum, the CONP's technical infrastructure allows it to operate as a unique hybrid between a traditional centralized data repository and a decentralized discovery tool that operates across multiple distinct repositories. The stewardship practices of the CONP align with and support its choice of technical architecture, enables centralized hosting and access to commonly permissioned data, and discovery and download of externally hosted and distinctly permissioned data. This achieves a compromise between competing policy imperatives: incentivizing data contributors to adopt similar data conditions of data governance, and enabling data that is not subject to harmonised data governance conditions to be searched for through a singular data discovery platform [11].

Part Two: Conceptualizing data governance practices for open access data sharing

The shift from holding coded human biomedical data in controlled access to full open access requires a correlative change in the governance measures that are used to safeguard the rights and interests of individuals who consent to have their data be hosted in the concerned repositories.

Declining costs in information processing, data storage, and data analysis have made viable large-scale, data-intensive biomedical research that leverages existing data from multiple repositories. This has produced a concomitant shift in the practical application of international biomedical research ethics principles. In the past, informed consent materials and data governance practices restricted the use of research data to the research project in which they were generated, and narrowly limited reuse to other closely related research efforts [12]. This reflected the high costs of and limited technical prospect for repurposing data outside of the research project for which it was originally generated. As the cost-effective aggregation of large quantities of biomedical research data became technically possible, tools and career specializations to support data interoperability proliferated (e.g., dedicated personnel trained to

perform data harmonization and data management, stakeholder forums dedicated to elaborating and refining shared ontologies, file formats, and other interoperability standards) [13; 14; 15]. Corresponding changes in biomedical research ethics practice and data sharing policies followed.

International biomedical research ethics instruments, such as the World Medical Association Declaration of Taipei, increasingly legitimate the indefinite storage of biomedical research data in databases and repositories for long-term reuse. Research communities have started to shift from obtaining purpose-limited and time-limited consent, to securing broad consent to the ongoing use of data, conditional on its continued stewardship [16]. This governance approach is often realized through the use of a controlled-access mechanism, through which researchers obtain informed consent or other authorizations to remove the most conspicuous individual identifiers from the data to mitigate privacy risks (i.e., the data is coded) and subsequently deposit the de-identified data in repositories for their long-term retention and future use. Data stewards bring together relevant bioethics and legal expertise, scientific knowledge of the concerned field of research, and technical skills relevant to the operation and management of the host database. These actors perform oversight and devise general policies that determine how that data can be used. Accredited researchers can then submit applications to these governance bodies for access to data for a specified period of time and for a specified purpose.

Controlled access mechanisms leverage data de-identification and ongoing control of downstream data use to minimize the residual risks of privacy infringement and information misuse to almost nil. The administrative burdens associated with the submission and oversight of data access requests, however, can preclude researchers from performing the scalable use of data across multiple biorepositories, as well as greatly reducing the likelihood of wider and deeper exploration of valuable existing data by the scientific community. Furthermore, because instituting and maintaining access committees is labor- and resource-intensive, the choice to hold data according to controlled access can create challenges for the long-term financial and operational sustenance of such repositories [17].

Navigating the controlled access processes to obtain access to data creates considerable administrative burdens for researchers, especially those in low-and-middle-income countries or outside traditional academic research organizations (e.g., SMEs or citizen scientists) [18; 19]. Relative to controlled access, the deposit of data in full open-access offers a number of benefits for researchers, publics, and research participants. Many research participants and communities of research participants express a desire to share their data in full open access.

Though the sharing of data, subject to controlled-access mechanisms, and other restrictions on data use, will remain a standard data stewardship practice for the foreseeable future, demand on the part of researchers and research participants, and the increasing benefits of data use at scale, favor the creation of data repositories dedicated to sharing biomedical research data in full open access as a default practice. The greatest challenge associated thereto is to create data stewardship processes that are suitable to data that will be held for long-term future use in open, public repositories, for which no oversight of the case-specific uses made of open access data can be performed.

The CONP enables full open access to research data by leveraging participants' informed consent and the use of data de-identification requirements to further mitigate the risk of individual re-identification or harmful data use. This shifts the core of the governance approach from post-ingestion active stewardship to rigorous pre-ingestion informed consent and data de-identification.

The CONP Ethics and Data Governance Committee

The CONP Ethics and Governance Committee, composed of experts in bioethics, neuroethics, and law, has produced a CONP Governance Framework [20] that establishes the central concepts and principles that inform CONP governance policies, as well as guidance to translate these principles into immediate practice and long-term objectives for the governance of open data that require additional deliberation to implement. The Framework's guiding principles are: (i) researcher integrity, (ii) autonomy, (iii) privacy, (iv) scope of data access and use, (v) capacity to consent, (vi) participant health, (vii) community engagement, and (viii) trust and accountability. The Governance Framework incorporates detailed sub-

points articulating each principle and translating them into applicable rules or expanding upon the values that each reflects.

Drawing from the Governance Framework, the CONP has developed Consent and Privacy and De-identification guides detailing ethics and data governance requirements that apply to prospective data contributors. Together, these three documents form the CONP Ethics Toolkit. As discussed above, respect thereof is required for data contributors that store their data on local CONP digital infrastructure. The CONP has further innovated in creating metadata elements that can be associated to datasets to describe the conditions of use associated thereto in a manner that will trail the data as it is downloaded from the CONP. Below, we describe these elements in detail. In addition to performing the foregoing functions, the Ethics and Data Governance Committee provides ad-hoc guidance to the operational staff of the CONP, to help it respond to governance challenges as they arise. This includes tailoring the CONP guidance tools to respond to new risks or requirements and providing counsel on their application to specific factual circumstances.

The CONP Consent Guide

Obtaining informed consent is a precursor to performing scientific research involving human participants. The information provided to participants during the informed consent process often determines the conditions according to which acquired data can be used for future research purposes. Re-using data in a manner outside the scope of an existing informed consent often requires considerable investment in either obtaining confirmation from a research ethics board (REB) that it is ethical to proceed absent a new consent or seeking a new informed consent from the concerned individuals.

The CONP Consent Guide provides guidance for researchers obtaining informed consent to the collection of data for the purpose of submitting it to the CONP Portal’s Community Server or in determining whether an existing informed consent is suitable for such a submission. It has three main components. First, it contains a list of core consent elements that must be reflected in the informed consent materials of research studies that intend to contribute data to the CONP Portal’s Community Server. Second, the

Guide contains a retrospective consent filter. This is a self-assessment tool that enables researchers to determine if they have included the necessary elements for open data sharing in their study's Informed Consent Form (ICF) and their data can be contributed to the Community Server as-is, or if additional steps might be required before such data is suitable for contribution to the CONP. Other biomedical research consortia, such as the Human Cell Atlas (HCA) and the International Cancer Genome Consortium (ICGC), have used retrospective consent filters to guide researchers in depositing data in open access [21]. Third, the CONP Consent Guide also contains template clauses that reflect the foregoing core consent elements, which researchers can adapt to meet local regulatory requirements or institutional demands.

The CONP Core Consent Elements are as follows:

1. Generation of participant data for research purposes.
2. Data de-identification (i.e., coding, anonymization, or synthetic data generation).
3. Sharing of de-identified data via the CONP Portal, an open-access platform that researchers the world over may access.
4. De-identified data can be used for commercial purposes.
5. Not possible to withdraw data that has already been shared.
6. Low risk that the participant could be re-identified in the future.

These core consent elements contain broad permissions which allow data to be stored in open platforms that scientists and the general public can use for research purposes without imposing major limitations on how data may be used. Further, the information provided enables research participants to understand the risks inherent to their data being used and to appreciate the limits on a potential withdrawal of the submitted data. These elements are derived from the generalist clauses of the Global Alliance for Genomics and Health (GA4GH) and, therefore, can be used in a manner that is interoperable with other data that have been collected according to GA4GH standards or close derivatives thereof [22]. This approach builds on the implementation of broad consent to data sharing in other large-scale biomedical

research consortia, leveraging appropriate risk disclosure, consent to broad data sharing, and data de-identification to disclose and mitigate the potential privacy risks associated to data sharing [16]. The governance strategy of the CONP consists in using risk disclosures and data de-identification to communicate and mitigate risks of individual re-identification, rather than performing ongoing governance of data access requests. The foregoing consent guidance therefore requires researchers to inform the research participants that their data will be shared with the public to enable its open research there remains a small residual risk that their data could be re-identified in the future. In contrast to the pairing of broad consent and use-specific access controls, the CONP's approach to data governance emphasizes risk communication and data de-identification as its principal data stewardship mechanisms.

The CONP Privacy and De-identification Guide

The de-identification of data can often be an ethical or legal precondition to its continued use or its transmission to third parties. De-identification is a context-specific procedure that requires data stewards to remove or transform (e.g., generalize) the features of a dataset that could enable individual re-identification and those that are highly sensitive and potentially detrimental to the individual. The CONP has developed a Privacy and De-identification Guide that helps researchers establish how data should be de-identified prior to its submission to the CONP Portal's Community Server. It requires data contributors to reduce the risk of individual re-identification to a low residual likelihood of re-identification prior to submitting such data to the CONP for public disclosure. This guide is also intended to propose standard mechanisms for assessing data identifiability and for performing data de-identification that other open neuroscience communities can adopt. It restates key concepts from Canadian research ethics guidance, from the regulatory guidance of Canadian privacy commissioners, and concepts established in data protection law.

To help scientists reduce the risk of individual re-identification as much as possible while maintaining the scientific utility of the data, CONP Privacy and De-identification Guide provides links to resources that are tailored to neuroscience data, including user-facing tools such as de-identification guidance or

algorithms that remove identifying information (e.g., names or birthdates from data file headers) or features of the data (e.g., facial features from magnetic resonance images). It also recommends tools that enable the generation of synthetic data and help researchers assess whether their data are best held in controlled access, registered access, or open access repositories according to their sensitivity and associated risk of re-identification [23].

Ethics Provenance Metadata and the Data Upload Process

Platforms that host data for secondary use are required to communicate to data contributors their responsibility to obtain required authorizations prior to depositing data and for compliance with the platform's data submission policies. More onerous methods of managing data submission include the use of contracts and data contribution forms that are subject to expert review prior to the upload of data to a platform, sometimes requiring attestations and signatures from authoritative institutional representatives of the submitting institution. Less onerous mechanisms include the use of 'click-wrap' agreements that require data contributors to assert their understanding of and compliance with the preconditions of data submission, which pop-up on the screen of the contributor as part of the data submission process [24]. The data upload form requires 1) an attestation that one of four acceptable conditions for data upload has been satisfied[1], 2) the parties uploading the data to specify which open intellectual property license has been applied to their data, and 3) uploaders to stipulate whether the data is held in open access, registered access, or controlled access. For data hosted directly on the CONP's technical infrastructure, open access is currently the only permissible choice in this respect. Last, for those datasets that attest that a Research Ethics Board has performed the oversight of their data, the applicable REB approval number is also

[1] These include: (1) participants have provided a valid informed consent to the de-identification and deposit of their data in an open-access portal; or (2) a waiver or other authorization to deposit these de-identified data in an open-access portal was obtained from a research ethics body (REB, IRB, REC, etc.); or (3) local law or a relevant institutional authorization otherwise enables the deposit of these data in an open-access portal; or, (4) these data are not derived from human participants.

uploaded, thereby providing a measure of evidence that their data has genuinely been subject to a REB evaluation.

The CONP has uses metadata elements that are integrated to the data upload form to collect information about the conditions of use applicable to uploaded data, and to ensure that the preconditions to hosting data on the CONP Portal are satisfied. Through this data upload form, data contributors are required to upload a minimal set of metadata (implemented in the form of the standard Data Tags Suite model) [25] when preparing a submission to the Portal. This requires researchers to specify information about the governance measures applied to their data as a precondition to being able to upload, actively prompting them to hold themselves accountable for their use of data.

Conclusion

The CONP's data governance policies and tools emphasize pre-submission informed consent practices, robust data de-identification tools, and the inclusion of ethico-legal metadata with shared data. The CONP therefore enables researchers to submit datasets in full open-access in compliance with their ethical, legal, and institutional commitments. This allows for increased pluralism in approaches to data stewardship represented amongst biomedical data repositories. Its approach provides a greater range of options to research participants and researchers in selecting the combination of data access controls, de-identification practices, and community rules that best align with their preferences and the ethical and legal commitments of their local institution. It is hoped that the CONP's approach to data stewardship might also serve as a model for other open neuroscience initiatives in Canada and elsewhere.

Declarations:

Ethics approval: Not applicable.

Consent for publication: Not applicable.

Data availability: Not applicable.

Competing interests: The authors have no competing interests to declare.

Centre of Genomics and Policy

Contributions: AB drafted the original manuscript. AB BMK PB MB AT AE conceptualized the manuscript. AB BMK PB MB AT AE drafted the policies and developed the tools described in the article. BMK PB BM AT AE provided comment on the manuscript and drafted sections of the amended and final manuscript. BMK AE acquired the funding used to produce the manuscript, and provided the resources required to deliver the described tools.

Funding: This manuscript was funded by a Brain Canada Platform Support Grant Competition Award in addition to funds and in-kind support from sponsor organizations.

References

1 Organisation for Economic Cooperation and Development (OECD). Open Science: Enabling Data Discovery in the Digital Age. Going Digital Toolkit Note, No. 13. 2021.

2 Organisation for Economic Cooperation and Development (OECD). Recommendation of the Council concerning Access to Research Data from Public Funding. C(2021)2. 2021.

3. United Nations Economic, Social, and Cultural Organisation (UNESCO). UNESCO Recommendation on Open Science. 2021.

4. Gilmore RO, Xu M, Adolph KE. Data sharing. In: Panicker S, Stanley B, editors. Handbook of research ethics in psychological science; 2021. American Psychological Association. p. 83–97.

5. Regev A, Teichmann S, Rozenblatt-Rosen O, Stubbington M, Ardlie K, Amit I et al. The Human Cell Atlas White Paper. 2018.

6. Ball MP, Bobe JR, Chou MF, Clegg T, Estep PW, Lunshof JE et al. Harvard Personal Genome Project: lessons from participatory public research. Genome Medicine 2014; 6:10.

7. Sneddon TP, Li P, Edmunds SC. GigaDB: announcing the GigaScience database. GigaScience 2012; 1:1.

8. Halchenko Y, Meyer K, Poldrack B, Solanky D, Wagner A, Gors J et al. DataLad: Distributed System for Joint Management of Code, Data, and Their Relationship. Journal of Open Source Software 2021 6:63, 3262.

9. Sharif T, Rioux P, Rousseau M, Kassis N, Beck N, Adalat R, et al. CBRAIN: A Web-Based, Distributed Computing Platform for Collaborative Neuroimaging Research. Frontiers in Neuroinformatics 2014; 8:54.

10. Beauvais MJS, Illes J, Knoppers BM. A Marathon, Not a Sprint - Neuroimaging, Open Science and Ethics. Neuroimage. 2021; 1:236:118041.

11. Thorogood A. Policy-Aware Data Lakes: A Flexible Approach to Achieve Legal Interoperability for Global Research Collaborations. Journal of Law and the Biosciences, 2020; 7:1.

12. Bernier A, Knoppers BM. Longitudinal Health Studies: Secondary Uses Serving the Future. Biopreservation and Biobanking. 2021; 19:5 404-413

13. Luo J, Wu M, Gopukumar D & Zhao Y. Big data application in biomedical research and health care: a literature review. Biomedical informatics insights. 2016; 8:BII-S31559.

14. Navale V, Bourne PE. Cloud Computing Applications for Biomedical Science: A Perspective. PLoS Comput Biol 208; 14:6: e1006144.

15. Cirillo D, Valencia A. Big data analytics for personalized medicine. Current opinion in biotechnology. 2019; 58:161-167.

16. Boers SN, van Delden JJN, Bredenoord AL. Broad Consent is Consent for Governance. The American Journal of Bioethics. 2015; 15:9 53-55.

17. Devriendt T, Borry P, Shabani M. Factors that influence data sharing through data sharing platforms: A qualitative study on the views and experiences of cohort holders and platform developers. *PLOS One* 2021; 16:7: e0254202.

18. Corpas M, Kovalevskaya NV, McMurray A, Nielsen FG. A FAIR Guide for Data Providers to Maximise Sharing of Human Genomic Data. PLoS Comput Biol 2018; 14:3: e1005873.

19. Shabani M, Borry P. "You want the right amount of oversight": interviews with data access committee members and experts on genomic data access. Genet Med 2016; 18: 892–897.

20. Cavoukian C et al. CONP Ethics and Data Governance Framework. Canadian Open Neuroscience Platform, 2019.

21. Wallace SE, Kirby E, Knoppers BM. How can we not Waste Legacy Genomic Data? Front Genet. 2020; 11:446.

22. Global Alliance for Genomics and Health. Consent Clauses for Genomic Research. Global Alliance for Genomics and Health Regulatory and Ethics Toolkit. 2020.

23. Knoppers BM, Beauvais MJS, Cavoukian A, Clarkson J, Green-Noble L, Illes J et al. Canadian Open Neuroscience Platform (CONP) Ethics Toolkit. 2022.

24. Bernier A, Molnár-Gábor F, Knoppers BM. The International Data Governance Landscape. Journal of Law and the Biosciences 2022; 9:1 :lsac005.

25. Sansone SA, Gonzalez-Beltran A, Rocca-Serra P, Alter G, Grethe JS, Xu H et al. DATS, the data tag suite to enable discoverability of datasets. Sci Data 2017; 4:170059.
