# Supplementary material for: Open Data Governance at the Canadian Open Neuroscience Platform (CONP): From the Walled Garden to the Arboretum
Source: Gigascience. 2024 Jan 13;13:giad114. doi: 10.1093/gigascience/giad114 (PMC10787360; doi:10.1093/gigascience/giad114)
Supplement: giad114_GIGA-D-23-00204_Revision_1 [file giad114_giga-d-23-00204_revision_1.pdf]

GigaScience

Open Data Governance at the Canadian Open Neuroscience Platform (CONP): From the Walled Garden to the Arboretum

--Manuscript Draft--

Manuscript Number:	GIGA-D-23-00204R1
Full Title:	Open Data Governance at the Canadian Open Neuroscience Platform (CONP): From the Walled Garden to the Arboretum
Article Type:	Review
Funding Information:	Fondation Brain Canada / Prof Alan Evans
Abstract:	Scientific research communities pursue dual imperatives in implementing strategies to share their data. These communities attempt to maximize the accessibility of biomedical data for downstream research use, in furtherance of open science objectives. Simultaneously, such communities safeguard the interests of research participants through data stewardship measures and the integration of suitable risk disclosures to the informed consent process. The Canadian Open Neuroscience Platform (CONP) convened an Ethics and Governance Committee composed of experts in bioethics, neuroethics, and law, to develop holistic policy tools, organisational approaches, and technological supports to align the open governance of data to ethical and legal norms. The CONP has adopted novel platform governance methods that favor full data openness, legitimated through the use of robust de-identification processes and informed consent practices. The experience of the CONP is articulated as a potential template for other open science efforts to further build upon. This experience highlights informed consent guidance, de-identification practices, ethico-legal metadata, platform-level norms, and commercialization and publication policies as the principal pillars of a practicable approach to the governance of open data. The governance approach which the CONP has adopted for its open data stands as a viable model for the broader open science community, and broader neuroscience community, to adopt in sharing data in full open access.
Corresponding Author:	Alexander Bernier, J.D. LL.M.<br>University of Toronto Faculty of Law<br>Toronto, CANADA
Corresponding Author Secondary Information:	
Corresponding Author's Institution:	University of Toronto Faculty of Law
Corresponding Author's Secondary Institution:	
First Author:	Alexander Bernier, J.D. LL.M.
First Author Secondary Information:	
Order of Authors:	Alexander Bernier, J.D. LL.M.
	Bartha M. Knoppers, PhD
	Patrick Bermudez, PhD
	Michael J. S. Beauvais, BCL JD
	Adrian Thorogood, BCL LLB LLM
	CONP Consortium†
	Alan Evans, PhD
Order of Authors Secondary Information:	
Response to Reviewers:	Response to peer reviewers for the manuscript entitled “Open Data Governance at the Canadian Open Neuroscience Platform (CONP): From the Walled Garden to the Arboretum” (manuscript number: GIGA-D-23-00204)

Powered by Editorial Manager® and ProduXion Manager® from Aries Systems Corporation

We sincerely thank the reviewers for their thoughtful and in-depth contributions to the development of this manuscript.

Reviewer 1:

Comment:
However, I will absolutely under no circumstances accept this point made by the authors about how metadata may be kept

"...and the GitHub open software repository to host the dataset metadata"

The authors seem to believe erroneously that GitHub is an open software repository that can permanently host dataset metadata. While this may be relatively true for pair repositories this is not generally true. They fail to understand that this open software repository is not persistent. They may not recommend ANYWHERE (manuscript or on their website) the use of GitHub as a repository! GitHub is owned by Microsoft and removes or eliminates repositories that are no longer actively developed much to the dismay of many grad students and postdocs who have had to beg Microsoft to bring back their data, with little success. The only way to guarantee anything from GitHub is to push a copy of the repo to Zenodo. Unless the policy at GitHub has recently changed, which I would need evidence for, this point must be clearly addressed.

Response:
We understand and are sensitive to the concern the reviewer raises here. In addressing it, we first offer the following points of clarification:
1)Our text makes no claims or suggestions as to the permanence of data hosted on GitHub and, indeed, it would be silly to claim the permanence of any store, whether commercial or institutional. Like any repository, we have policies and practices for best-effort preservation which we adhere to seriously. As is the case for identifiers such as DOIs and the objects they point to, we should rather speak of persistence and not permanence. In our examination (which we believe is consistent with that of the wider community), pragmatically speaking, GitHub is not meaningfully less persistent a resource than others. We did not undertake this design choice lightly or out of expedience but with full consideration of the role GitHub has come to occupy in the research community, though we agree there remain live issues concerning how the community as a whole uses GitHub.
2)The CONP Portal does not rely exclusively on GitHub to store dataset metadata. Indeed, the copy of each dataset's metadata (in the form of a "DATS.json" file) that is available on the Portal is hosted on the CONP's local servers and not GitHub.
3)Though we were unable to find a stated policy of removing inactive repositories, and research into community experiences only revealed deletion under unusual circumstances (e.g., serious violation of policy or loss of access to original repo), we are concerned by the reviewer's experience with GitHub and thank them for this relevant warning. We will further investigate the conditions under which GitHub manages low-activity repositories, the rare conditions under which a deletion might occur, and the warning provided to users in such cases.
4)Though GitHub does not seem any less reliable than typical institutional stores, we again emphasize that the exceedingly unlikely deletion of a dataset's GitHub-hosted metadata would require intervention from the CONP's staff to restore updatability and version tracking but it would not affect the availability of the dataset or its metadata to the community as offered through the CONP Portal.

A footnote has been added to the manuscript as a point of clarification:
"Metadata are also stored locally on the CONP Portal's servers and accompany every dataset, whether through browser-based access or through DataLad."

Comment:
A major omission of this work is also in the lack of discussion of the role of INCF in policy, standards setting and the contribution of this important international organization in this space. As far as I understand it, the CONP took significant work from INCF and enhanced it, but this manuscript does not address the role that INCF has played.

Powered by Editorial Manager® and ProduXion Manager® from Aries Systems Corporation

Response:
The CONP Principal Investigator and the CONP personnel responsible for the ethics and governance portfolio of the platform throughout its lifetime have been consulted to address this issue. Based on these conversations, our understanding of the relationship between the INCF and the CONP is the following.

The INCF has been engaged in significant standard-development efforts in a number of areas related to neuroinfomatics, which, indeed, is among the reasons the CONP, from its very inception, established a fruitful partnership and collaboration with the INCF. Having reviewed the history of the development of the CONP's Ethics & Governance policies presented in this manuscript with the personnel responsible for its elaboration, to the knowledge of the authors, the CONP has not directly used prior technical or governance approaches that the INCF developed as the basis of its own technologies and practices. Many of the CONP's approaches are founded chiefly on the work of Global Alliance for Genomics and Health.

Needless to say, the CONP does not operate in a vacuum and it was certainly not the intent of the authors to passively diminish the importance of any contributors to this space, one to which the CONP itself wishes to contribute. Some text has been added to acknowledge the INCF's contributions alongside a few other entities. The relevant sections now reads:

"The Canadian Open Neuroscience Platform (CONP) is among an increasing number of international initiatives working to develop policy standards for the open and unrestricted sharing of human biomedical research data. Other examples of organizations who have fostered the development of policies, standards, and tools that facilitate the interoperable sharing of neuroscience data include the Personal Genome Project, the GigaScience GigaDB, the Human Cell Atlas, the Global Alliance for Genomics and Health (GA4GH), and the International Neuroinformatics Coordinating Facility (INCF) [5, 6, 7, 8, 9, 10, 11, 12]."

Comment:
On a more minor point, it would be useful if CONP would create some sort of ontology based search or at least would use a standard lemenalization library in their search box. I searched for Alzheimers and got 0 results while Alzheimer brought back several studies. That omission of simple search technologies is simply unacceptable in the age of google, and Chat bots.

Response:
We very much agree that the CONP Portal's search functionality needs improvement and feature extension, including ontology-based search as the reviewer suggests. Such features are among the Portal's technical roadmap items we hope to implement over the next year of development work.

Reviewer 2:

Comment:
My only concern is the manner in which CONP handles complex cases.

Some countries may have restrictions on using data outside of a clinic for the purpose of sharing, while others may have no specific legal regulation on data ownership, albeit with administrative rules that would lead to a situation in which there are no declared boundaries or rules to be followed when sharing data. In nations where the majority of clinical data are collected in state-owned hospitals, data sharing may be difficult. Does CONP facilitate a mechanism that not only enables the sharing of current data but also encourages the sharing of potential data in an effort to increase the volume of such data? This may increase the need for a committee to supervise the platform, evaluate data submissions, and, most importantly, provide direction on data sharing procedures. In addition, since there are already clear and straightforward procedures (such as self-checklists), difficult cases requiring assistance from more specialists would be eliminated from the start. In other words, some researchers would be irritated by the

Powered by Editorial Manager® and ProduXion Manager® from Aries Systems Corporation

clear-cut standard and somewhat automated processing of CONP. (such as self-checklist), which would eliminate difficult cases that require the assistance of more specialists.

Response:
These considerations are indeed quite valid. The design of the CONP governance approach is not intended to displace or replace the more fulsome approaches to governance that other platforms have integrated. Rather – it is intentionally designed to foster pluralism in the governance and stewardship conditions that can be applied to data through the platform. To this end, datasets that require more specialised approaches to governance can be either uploaded to other repositories that have implemented the required stewardship measures and can be made discoverable and accessible through the CONP Portal or (in specific cases that continue to broaden with the Portal's technical development) through credentialed access to data stored in a specific data-management system (e.g., the LORIS platform) which nonetheless allow access through the Portal. These are currently available options for datasets that require more thorough governance and access control.

Further to this, the CONP regularly engages in open dialog with potential data contributors to help evaluate and plan both retrospective and prospective data contributions that account for the governance obligations of both parties. The latter case often involves advice concerning the elements of informed consent and data anonymization the CONP recommends generally and, in the case where CONP servers are used to host data, are required. In this way, we hope to encourage broader open-science practice through some consideration of the possibility of open data-sharing as early as possible in the planning stages of a study. Doing so greatly facilitates matters down the line. The types of interactions with the community feed back into the ongoing work of CONP's Ethics & Governance committee and its endeavour to respond to questions regarding data deposit or governance. In some cases, these efforts may take the form of iterative dialogues that result in bespoke safeguards or paths to data deposit for specific communities.

To clarify these nuances in the text, we have added certain elements to the manuscript (changes are highlighted in bold):

"In addition to performing the foregoing functions, the Ethics and Data Governance Committee provides ad-hoc guidance to the operational staff of the CONP and to researchers who intend to deposit data on the CONP Portal, responding to governance challenges as these arise. This includes tailoring the CONP guidance tools to respond to new risks or requirements and providing counsel on their application. The Ethics and Data Governance Committee further monitors for potential risks associated to the upload of specific categories of data, specific populations, or circumstances of data upload that might require the imposition of additional governance controls on a contextual basis, especially where communities indicate that data pertaining to their members requires additional governance safeguards to mitigate the potential for public data dissemination to lead to group-level harms (15, 26, 27)."

Reviewer 3:

Comments (not replicated here in full – but have addressed them in the comments below):

Response:
The considerations raised throughout this peer review are deeply perceptive, forward-looking, and rich with detail. The prospective alignment of consent practices and de-identification processes to those of the repositories in which data are anticipated to be deposited is a common precondition to data contribution. Repositories define their consent and identifiability requirements as a matter of habitual course.

The heightened emphasis that the manuscript places on the foregoing governance safeguards is not intended to suggest that individual-facing safeguards such as data de-identification and consent are the sole mechanisms that should be implemented to safeguard research participants against harms, or that group harms should not be

safeguarded against. Defining consent and de-identification practices is accorded great importance in bioethics literature generally, and especially in the binding Research Ethics requirements detailed in the TCPS-2. One of the principal aims of this manuscript is to detail how repositories can interface with the consent and de-identification requirements that are applicable to researchers in depositing data in such repositories, so as to help researchers prepare their data for the release of data in full open-access and determine when this is appropriate. For this reason, a great deal of the word-count has been dedicated to addressing these issues.

The mitigation of group harms – and addressing population-specific concerns – often occurs through the engagement of the Research Ethics Committees (RECs) in the oversight processes that are dedicated to individual studies. Because this occurs prior to the deposit of data in repositories, it is not addressed at the stage of administering the contribution of data to repositories.

The manuscript is directed to establishing practicable approaches to sharing data that have been consented and de-identified in a manner that renders its open release lawful and compliant with research ethics guidance. Much of the test is therefore used to explore and outline the approaches that the CONP has at present adopted to enable oversight – at the level of the repository – of data which is intended for sharing in full open access.

Finding practical mechanisms to manage and to address potential group harms or population-specific risks and data governance practices is nonetheless an extremely important endeavor, one in which the CONP is also engaged.

Text has been added to this manuscript stating that the CONP Ethics and Data Governance Committee is devising specialised governance controls for specific populations that act as additional safeguards to deposit their data in full open access, and emphasizes the importance thereof to making open science more inclusive and equitable:

The first section of this text elaborates the important role of Research Ethics Committees and other stakeholders in addressing these categories of concerns:

"The CONP governance model responds to prevailing legal paradigms and applicable ethics requirements, to enable the open sharing of neuroscience data consented or permissioned for open release. The mitigation of population-specific or group-specific harms is intermediated through the involvement of Research Ethics Committees (RECs) that oversee research or through other stakeholders, such as patient communities and population-specific research organizations, prior to the upload of individuals' coded data to the CONP. These actors mitigate such risks in overseeing the drafting of informed consent materials, determining how data can be collected, de-identified, and released, and assessing whether a particular data repository is suitable for the deposit of data. In the future, collaboration with stakeholders from relevant populations will be required to tailor the governance approach of the CONP to the risks that affect specific vulnerable groups, communities of patients with unique needs, and indigenous communities. This could require additional measures to tailor consent procedures to account for population-specific risks, for example to enable data contribution from those persons that do not have legal capacity to provide informed consent on their own behalf (e.g., pediatric populations, or patients that suffer from neurodegenerative diseases), or to implement population-specific de-identification or data manipulation processes that mitigate relevant group-level harms. This will further enable the dissemination of data relative to populations underrepresented in research datasets through the CONP."

As noted in the response to one of Reviewer 2's comments, text has been added to highlight that the CONP Ethics and Data Governance Committee further considers the implementation of population-specific or group-specific safeguards on an ongoing basis (as highlighted):

"In addition to performing the foregoing functions, the Ethics and Data Governance Committee provides ad-hoc guidance to the operational staff of the CONP and to researchers that intend to deposit data on the CONP, responding to governance

Powered by Editorial Manager® and ProduXion Manager® from Aries Systems Corporation

	challenges as these arise. This includes tailoring the CONP guidance tools to respond to new risks or requirements and providing counsel on their application to specific factual circumstances. The Ethics and Data Governance Committee further monitors for potential risks associated to the upload of specific categories of data, specific populations, or circumstances of data upload that might require the imposition of additional governance controls on a contextual basis, especially where communities indicate that data pertaining to their members requires additional governance safeguards to mitigate the potential for public data dissemination to lead to group-level harms (15; 26; 27)."<br><br>In addition to the incorporation of this text – the CONP is experimenting with a number of additional safeguards at this time. These are not listed in the manuscript as their design and implementation are still in their early phases.<br><br>One further note is that Figure 1 has been added to manuscript to further improve the clarity of its presentation of the governance models available to researchers that deposit data in the CONP.
Additional Information:	
**Question**	**Response**
Are you submitting this manuscript to a special series or article collection?	No
**Experimental design and statistics**<br><br>Full details of the experimental design and statistical methods used should be given in the Methods section, as detailed in our Minimum Standards Reporting Checklist. Information essential to interpreting the data presented should be made available in the figure legends.<br><br>Have you included all the information requested in your manuscript?	Yes
**Resources**<br><br>A description of all resources used, including antibodies, cell lines, animals and software tools, with enough information to allow them to be uniquely identified, should be included in the Methods section. Authors are strongly encouraged to cite Research Resource Identifiers (RRIDs) for antibodies, model organisms and tools, where possible.<br><br>Have you included the information requested as detailed in our Minimum Standards Reporting Checklist?	Yes

**Availability of data and materials**<br><br>All datasets and code on which the conclusions of the paper rely must be either included in your submission or deposited in publicly available repositories (where available and ethically appropriate), referencing such data using a unique identifier in the references and in the "Availability of Data and Materials" section of your manuscript.<br><br>Have you have met the above requirement as detailed in our Minimum Standards Reporting Checklist?	Yes

Manuscript Click here to access/download;Manuscript;final manuscript file.docx

Open Data Governance at the Canadian Open Neuroscience Platform (CONP): From the Walled Garden to the Arboretum

Alexander Bernier[1], Bartha M. Knoppers,[1] Patrick Bermudez[2], Michael J. S. Beauvais[3], Adrian Thorogood [4], CONP Consortium[†], Alan Evans[2].

[1] Centre of Genomics and Policy, Department of Human Genetics, Faculty of Medicine and Health Sciences, McGill University, Montréal, Québec, Canada

[2] McGill Centre for Integrative Neuroscience, Montreal Neurological Institute, McGill University, Montréal, Québec, Canada

[3] Faculty of Law, University of Toronto, Toronto, Ontario, Canada

[4] The Terry Fox Research Institute, Montreal, Quebec, Canada

[†] CONP Consortium (listed alphabetically): Brendan Behan, Pierre Bellec, Shawn Brown, David Bujold, Ann Cavoukian, John Clarkson, Samir Das, Emilie Dessureault, Moyez Dharsee, Erin Dickie, Simon Duchesne, Stephanie Dyke, Ken Evans, Alan Evans, Jennifer Flynn, Nils Forkert, Tom Gee, Tristan Glatard, Richard Gold, Rachel Harding, Felipe Henriques, Sean Hill, Judy Illes, Jason Karamchandani, Ali Khan, Greg Kiar, Bartha Maria Knoppers, Xavier Lecours, Melanie Legault, Dave MacFarlane, Cécile Madjar, Roland Nadler, Santiago Paiva, Paul Pavlidis, Jean-Baptiste Poline, David Rotenberg, Marc-Etienne Rousseau, Walter Stewart, Nikola Stikov, Elizabeth Theriault.

Abstract: Scientific research communities pursue dual imperatives in implementing strategies to share their data. These communities attempt to maximize the accessibility of biomedical data for downstream research use, in furtherance of open science objectives. Simultaneously, such communities safeguard the interests of research participants through data stewardship measures and the integration of suitable risk disclosures to the informed consent process. The Canadian Open Neuroscience Platform (CONP) convened an Ethics and Governance Committee composed of experts in bioethics, neuroethics, and law, to develop holistic policy tools, organisational approaches, and technological supports to align the open governance of data with ethical and legal norms. The CONP has adopted novel platform governance methods that favor full data openness, legitimated through the use of robust de-identification processes and informed consent practices. The experience of the CONP is articulated as a potential template for other open science efforts to further build upon. This experience highlights informed consent guidance, de-identification practices, ethico-legal metadata, platform-level norms, and commercialization and publication policies as the principal pillars of a practicable approach to the governance of open data. The governance approach adopted by the CONP stands as a viable model for the broader neuroscience and open science communities to adopt for sharing data in full open access.

Introduction

Open Science promotes the open dissemination of data, software, materials, manuscripts, and other outputs of scientific research to make them more transparent, accessible, and reproducible. A broad cross-section of international bodies, including the OECD and UNESCO, have recognized the potential for open science to benefit both the general public and scientific research communities [1, 2, 3]. The justifications for open science practices are wide-ranging and mutli-dimensional, and remain subject to ongoing community debate and elaboration. Oft-cited considerations include enabling public participation in defining research questions and performing scientific research, reducing the barriers to accessing research materials, and ensuring scientific accountability.

The siloed storage of biomedical research data hinders the pursuit of accessible, inclusive, and reproducible research. Greater openness in the sharing of data enables community-wide collaboration to improve the reproducibility of findings, conduct large-scale agglomeration of data that enhance statistical power, and improve the representation of underserved populations [4]. The Canadian Open Neuroscience Platform (CONP) is among an increasing number of international initiatives working to develop policy standards for the open and unrestricted sharing of human biomedical research data. Other examples of organizations that have fostered the development of policies, standards, and tools that facilitate the interoperable sharing of neuroscience data include the Personal Genome Project, the GigaScience GigaDB, the Human Cell Atlas, the Global Alliance for Genomics and Health (GA4GH), and the International Neuroinformatics Coordinating Facility (INCF) [5, 6, 7, 8, 9, 10, 11, 12]. The CONP has developed policies, practices, and technological tools for the open-access sharing of neuroscience data. Its approach rests on international bioethics norms, responds to regulatory requirements, and builds upon principles of open science, neuroethics, and privacy by design. The CONP emerged in Canada but it proposes approaches to open data sharing that can nonetheless be translated to other jurisdictions and other data types.

In recognition of the value of sharing results and lessons learned with a growing community that is facing similar and substantive challenges in developing and implementing open data-sharing practices, this

article details the open-data governance policies of the CONP and the tools that enable concordant practices. Part One describes the technical aspects of the CONP Portal (data- and tool-sharing infrastructure) and the premises underlying its submission policies. Part Two states the CONP's governance principles and the tools used to ensure that data submission is performed in a manner that respects established bioethics principles.

Part One: CONP data- and tool-sharing infrastructure and its governance

The CONP Portal is a purpose-built data- and tool-sharing platform that allows data to reside on different infrastructures through its flexible distributed management system. Portal users can choose among different methods of hosting their data, including third-party storage provided by the OSF, Zenodo, or storage native to the CONP technical infrastructure (via its 'Community Server'), and can benefit from even greater flexibility in data hosting location through the combined use of the DataLad distributed data-management system [13] and the GitHub open software repository to host the dataset metadata.[1] This enables the CONP to host data residing on both its own technical infrastructure and external data repositories, with the distinction being transparent to the user who can browse, search for, and access data irrespective of storage site. These design choices also give data depositors greater flexibility by not obligating them to upload their data to a single, centralized point, which may not be possible because of technical or legal impediments. Further features provide both browser-based and command-line access to data, as well as a pathway to high-performance computing via the CBRAIN interface [14].

The CONP Portal's technical design has implications for data stewardship. The CONP requires data contributors that host their data directly on CONP infrastructure to adhere to the stewardship standards that are detailed in its Consent Guide and its Privacy and De-Identification Guide [15]. Currently, CONP-hosted data are made available in full open access and are therefore available to all members of the public.

For data that are findable and accessible through the CONP Portal but not stored on its Community Server, it is sufficient for contributors to respect their local legal and biomedical research ethics requirements and the data stewardship policies of the selected host repository. In this latter case, adherence to the CONP data stewardship guidance is recommended but not required as, from a data governance standpoint, it is the stewardship practices of the host repository that ensure that externally hosted data are subject to appropriate oversight and it is unnecessary for the CONP to also mandate compliance with its own data stewardship practices. Conversely, the CONP requires adherence to its data governance standards for data it hosts natively and for which it therefore takes on the role of primary data steward.

This combination of technical and policy design enables the CONP to stipulate hosting conditions for all data residing on its own infrastructure while enabling external repositories to make their data discoverable and downloadable through the CONP, despite such data being held according to distinct data governance

[1] Metadata are also stored locally on the CONP Portal's servers and accompany every dataset, whether through browser-based access or through DataLad.

standards, including in registered or controlled access. This ensures that data that are useful for common research purposes can still be found and accessed through a singular data portal with the benefit of harmonized metadata.

In sum, the CONP's technical infrastructure allows it to span functionality that ranges between a traditional centralized data repository and a decentralized discovery tool that operates across multiple distinct repositories. The stewardship practices of the CONP align with and support its choice of technical architecture, enables centralized hosting and access to similarly permissioned data, and discovery and download of externally hosted and distinctly permissioned data (See Figure 1). This achieves a compromise between competing policy imperatives: incentivizing data contributors to adopt similar data conditions of data governance and enabling data that is not subject to harmonised data governance conditions to be discovered through a single platform [16].

Part Two: Conceptualizing data governance practices for open access data sharing

The shift from holding coded human biomedical data in controlled access to full open access requires a correlative change in the governance measures that are used to safeguard the rights and interests of individuals who consent to have their data hosted in the concerned repositories. Declining costs in information processing, data storage, and data analysis have made viable large-scale, data-intensive biomedical research that leverages existing data from multiple repositories. This has produced a concomitant shift in the practical application of international biomedical research ethics principles. In the past, informed consent materials and data governance practices restricted the use of research data to the research project in which they were generated, and narrowly limited reuse to other closely related research efforts [17]. This reflected the high costs of and limited technical prospect for repurposing data outside the research project for which it was originally generated. As the cost-effective aggregation of large quantities of biomedical research data became technically possible, tools and career specializations to support data interoperability proliferated (e.g., dedicated personnel trained to perform data harmonization and data management, stakeholder forums dedicated to elaborating and refining shared ontologies, file formats, and other interoperability standards) [18; 19; 20]. Corresponding changes in biomedical research ethics practice and data sharing policies followed.

International biomedical research ethics instruments, such as the World Medical Association's Declaration of Taipei, increasingly legitimate the indefinite storage of biomedical research data in databases and repositories for long-term reuse. Research communities have started to shift from obtaining purpose-limited and time-limited consent, to securing broad consent to the ongoing use of data, conditional on its continued stewardship [21]. This governance approach is often realized through the use of a controlled-access mechanism, through which researchers obtain informed consent or other authorizations to remove the most conspicuous individual identifiers from the data to mitigate privacy risks (i.e., the data is coded) and subsequently deposit the de-identified data in repositories for their long-term retention and future use. Data stewards bring together relevant bioethics and legal expertise, scientific knowledge of the concerned field of research, and technical skills relevant to the operation and management of the host database. These actors perform oversight and devise general policies that

determine how that data can be used. Accredited researchers can then submit applications to these governance bodies for access to data for a specified period of time and for a specified purpose.

Controlled access mechanisms leverage data de-identification and ongoing control of downstream data use to minimize the residual risks of privacy infringement and information misuse. The administrative burdens associated with the submission and oversight of data access requests, however, can prevent the scalable use of data across multiple biorepositories, as well as greatly reducing the likelihood of wider and deeper exploration of valuable existing data by the scientific community. Furthermore, because instituting and maintaining access committees is labor- and resource-intensive, the choice to hold data according to controlled access can create challenges for the long-term financial and operational sustenance of such repositories [22].

Navigating controlled access processes creates considerable administrative burdens for researchers, especially those in low-and-middle-income countries or outside traditional academic research organizations (e.g., SMEs or citizen scientists) [23, 24]. In decreasing the transaction costs inherent to accessing data, open access makes it possible for a broader community of researchers to perform studies with greater statistical power. Relative to controlled access, the deposit of data in full open access also aligns with the wishes of many research participants and communities to do so, maximizing their contribution to science. Open access may also hold the potential to reduce disparities in access to the benefits of research to the degree that it maximizes accessibility to data from underrepresented sub-populations.

Though the sharing of data subject to controlled access mechanisms and other restrictions on data use will remain a standard data stewardship practice for the foreseeable future, demand on the part of researchers and research participants, and the increasing benefits of data use at scale, favor the creation of data repositories dedicated to sharing biomedical research data in full open access as a default practice. The greatest challenge thereto is to create data stewardship processes that are suitable to data that will be held for long-term future use in open, public repositories, for which no oversight of the case-specific uses made of open access data can be performed.

The CONP enables full open access to research data by leveraging participants' informed consent and the use of data de-identification requirements to further mitigate the risk of individual re-identification or harmful data use. This shifts the core of the governance approach from post-ingestion active stewardship to rigorous pre-ingestion informed consent and data de-identification. This governance model responds to prevailing legal paradigms and applicable ethics requirements to enable the open sharing of neuroscience data that is consented or otherwise permissioned for open release. The mitigation of population-specific or group-specific harms is mediated through the involvement of Research Ethics Boards (REBs) that oversee research or through other stakeholders, such as patient communities and population-specific research organizations, prior to the upload of individuals' de-identified data to the CONP. These actors mitigate such risks in overseeing the drafting of informed consent materials, determining how data can be collected, de-identified, and released, and assessing whether a particular data repository is suitable for the deposit of data.

In the future, collaboration with stakeholders from relevant populations will be required for the CONP to tailor its governance approach to concerns that are specific to certain vulnerable groups or communities of patients with unique needs. For example, the CONP can seek to engage with indigenous communities to explore the degree to which open access approaches might align with community interests and data sovereignty. Population-specific governance modifications could include adjusting data governance and consent procedures to account for population-specific concerns, for example to enable data contribution from those who do not have legal capacity to provide informed consent on their own behalf (e.g., pediatric populations or patients that suffer from neurodegenerative diseases) or implementing population-specific de-identification or data manipulation processes that mitigate relevant group-level harms. This will further enable the dissemination of data relative to populations underrepresented in research datasets through the CONP Portal.

The CONP Ethics and Data Governance Committee

The CONP Ethics and Governance Committee, composed of experts in bioethics, neuroethics, and law, has produced a CONP Governance Framework [25] that establishes the central concepts and principles that inform CONP governance policies, as well as guidance to translate these principles into immediate practice and long-term objectives for the governance of open data that require additional deliberation to implement. The Framework's guiding principles are: (i) researcher integrity, (ii) autonomy, (iii) privacy, (iv) scope of data access and use, (v) capacity to consent, (vi) participant health, (vii) community engagement, and (viii) trust and accountability. The Governance Framework incorporates detailed sub-points articulating each principle and translating them into applicable rules or expanding upon the values that each reflects.

Drawing from the Governance Framework, the CONP has developed Consent and Privacy and De-identification guides detailing ethics and data governance requirements that apply to prospective data contributors. Together, these latter two documents form the CONP Ethics Toolkit. As discussed above, respect thereof is required for data contributors that store their data on CONP servers. The CONP has further innovated in creating metadata elements that can be associated to datasets to describe the conditions of use associated thereto in a manner that will trail the data as it is downloaded from the CONP. Below, we describe these elements in detail. In addition to performing the foregoing functions, the Ethics and Data Governance Committee provides *ad hoc* guidance to the operational staff of the CONP and to researchers who intend to deposit data on the CONP Portal, responding to governance challenges as these arise. This includes tailoring the CONP guidance tools to respond to new risks or requirements and providing counsel on their application. The Ethics and Data Governance Committee further monitors for potential risks associated to the upload of specific categories of data, specific populations, or circumstances of data upload that might require the imposition of additional governance controls on a contextual basis, especially where communities indicate that data pertaining to their members requires additional governance safeguards to mitigate the potential for public data dissemination to lead to group-level harms (15, 26, 27).

The CONP Consent Guide

Obtaining informed consent is a precursor to performing scientific research involving human participants and their identifiable data. The information provided to participants during the informed consent process often determines the conditions according to which acquired data can be used for future research purposes. Re-using data in a manner outside the scope of an existing informed consent often requires considerable investment in either obtaining confirmation from an REB that it is ethical to proceed absent a new consent or seeking a new informed consent from the concerned individuals.

The CONP Consent Guide provides guidance for researchers obtaining informed consent to the collection of data for the purpose of submitting it to the CONP Portal's Community Server or in determining whether an existing informed consent is suitable for such a submission. It has three main components. First, it contains a list of core consent elements that must be reflected in the informed consent materials of research studies that intend to contribute data to the CONP Portal's Community Server. Second, the Guide contains a retrospective consent filter. This is a self-assessment tool that enables researchers to determine whether they have included the necessary elements for open data sharing in their study's Informed Consent Form (ICF) and their data can be contributed to the Community Server as-is, or if additional steps might be required before such data is suitable for contribution to the CONP. Other biomedical research consortia, such as the Human Cell Atlas (HCA) and the International Cancer Genome Consortium (ICGC), have used retrospective consent filters to guide researchers in depositing data in open access [28]. Third, the CONP Consent Guide also contains template clauses that reflect the foregoing core consent elements, which researchers can adapt to meet local regulatory requirements or institutional demands.

The CONP Core Consent Elements are as follows:

1. Generation of participant data for research purposes.
2. Data de-identification (i.e., coding, anonymization, or synthetic data generation).
3. Sharing of de-identified data via the CONP Portal, an open-access platform that researchers the world over may access.
4. De-identified data can be used for commercial purposes.
5. Not possible to withdraw data that has already been shared
6. Low risk that the participant could be re-identified in the future.

These core consent elements contain broad permissions which allow data to be stored in open platforms that scientists and the general public can use for research purposes without imposing major limitations on how data may be used. Further, the information provided enables research participants to understand the risks inherent to their data being used and to appreciate the limits on a potential withdrawal of the submitted data. These elements are derived from the generalist clauses of the GA4GH and, therefore, can be used in a manner that is interoperable with other data that have been collected according to GA4GH standards or close derivatives thereof [29]. This approach builds on the implementation of broad consent to data sharing in other large-scale biomedical research consortia, leveraging appropriate risk disclosure, consent to broad data sharing, and data de-identification to disclose and mitigate the potential privacy risks associated to data sharing [21]. The governance strategy of the CONP consists in using risk disclosures and data de-identification to communicate and mitigate risks of individual re-identification, rather than performing ongoing governance of data access requests. The foregoing consent guidance therefore requires researchers to inform the research participants that their data will be shared with the

public to enable open research and that there remains a small residual risk that their data could be re-identified in the future. In contrast to the pairing of broad consent and use-specific access controls, the CONP's approach to data governance emphasizes risk communication and data de-identification as its principal data stewardship mechanisms.

The CONP Privacy and De-identification Guide

The de-identification of data can often be an ethical or legal precondition to its continued use or its transmission to third parties. De-identification is a context-specific procedure that requires data contributors or data stewards to remove or transform (e.g., generalize) the features of a dataset that could enable individual re-identification and those that are highly sensitive and potentially detrimental to the individual. The CONP has developed a Privacy and De-identification Guide that helps researchers establish how data should be de-identified prior to their submission to the CONP Portal's Community Server. It requires data contributors to reduce the risk of individual re-identification to a low residual likelihood prior to submitting data to the CONP for public disclosure. This guide is also intended to propose standard mechanisms for assessing data identifiability and for performing data de-identification that other open neuroscience communities can adopt. It restates key concepts from Canadian research ethics guidance, from the regulatory guidance of Canadian privacy commissioners, and concepts established in data protection law.

To help scientists reduce the risk of individual re-identification as much as possible while maintaining the scientific utility of the data, the CONP Privacy and De-identification Guide provides links to resources that are tailored to neuroscience data, including de-identification guidance or algorithms that remove identifying information, such as names or birthdates from data-file headers or facial features from magnetic resonance images. It also recommends tools that enable the generation of synthetic data and help researchers assess whether their data are best held in controlled access, registered access, or open access repositories according to their sensitivity and associated risk of re-identification [30].

Ethics Provenance Metadata and the Data Upload Process

Platforms that host data for secondary use are required to communicate to data contributors their responsibility to obtain required authorizations prior to depositing data and for compliance with the platform's data submission policies. More onerous methods of managing data submission include the use of contracts and data contribution forms that are subject to expert review prior to the upload of data to a platform, sometimes requiring attestations and signatures from authorized representatives of the submitting institution. Less onerous mechanisms include the use of 'click-wrap' agreements that require data contributors to assert their understanding of and compliance with the preconditions of data submission, which pop-up on the screen of the contributor as part of the data submission process [31].

The data upload form requires 1) an attestation that one of four acceptable conditions for data upload has been satisfied[2], 2) the parties uploading the data to specify which open intellectual property license has been applied to their data, and 3) uploaders to stipulate whether the data is held in open access, registered access, or controlled access. For data hosted directly on the CONP's technical infrastructure, open access is currently the only option. Last, for those datasets that attest that an REB has performed the oversight of their data, the applicable REB approval number is also provided as a measure of evidence that their data has genuinely been subject to an REB evaluation.

The CONP Portal data submission procedure requires data contributors to provide a minimal set of metadata (implemented in the form of the standard Data Tags Suite model) [32] along with their dataset. A subset of these metadata collect information about the conditions of use applicable to the data and ensure that the preconditions to hosting data on the CONP Portal are satisfied, thereby prompting contributors to hold themselves accountable for their use of data.

Conclusion

The CONP's data governance policies and tools emphasize pre-submission informed consent practices, robust data de-identification tools, and the inclusion of ethico-legal metadata with shared data. The CONP therefore enables researchers to submit datasets in full open-access in compliance with their ethical, legal, and institutional commitments. This allows for increased pluralism in approaches to data stewardship represented amongst biomedical data repositories. Its approach provides a greater range of options to research participants and researchers in selecting the combination of data access controls, de-identification practices, and community rules that best align with their preferences and the ethical and legal commitments of their local institution. It is hoped that the CONP's approach to data stewardship might also serve as a model for other open neuroscience initiatives in Canada and elsewhere.

Declarations:
Ethics approval: Not applicable.
Consent for publication: Not applicable.
Data availability: Not applicable.
Competing interests: The authors have no competing interests to declare.
Contributions: AB drafted the original manuscript. AB BMK PB MJSB AT AE conceptualized the manuscript. AB BMK PB MJSB AT AE drafted the policies and developed the tools described in the article. BMK PB MJSB AT AE provided comment on the manuscript and drafted sections of the amended and final manuscript. BMK AE acquired the funding used to produce the manuscript, and provided the resources required to deliver the described tools.

[2] These include: (1) participants have provided a valid informed consent to the de-identification and deposit of their data in an open-access portal; or (2) a waiver or other authorization to deposit these de-identified data in an open-access portal was obtained from a research ethics body (REB, IRB, REC, etc.); or (3) local law or a relevant institutional authorization otherwise enables the deposit of these data in an open-access portal; or, (4) these data are not derived from human participants.

Centre of Genomics and Policy

Funding: This manuscript was funded by a Brain Canada Platform Support Grant Competition Award in addition to funds and in-kind support from sponsor organizations.

References

1 Organisation for Economic Cooperation and Development (OECD). Open Science: Enabling Data Discovery in the Digital Age. Going Digital Toolkit Note, No. 13. 2021.

2 Organisation for Economic Cooperation and Development (OECD). Recommendation of the Council concerning Access to Research Data from Public Funding. C(2021)2. 2021.

3. United Nations Economic, Social, and Cultural Organisation (UNESCO). UNESCO Recommendation on Open Science. 2021.

4. Gilmore RO, Xu M, Adolph KE. Data sharing. In: Panicker S, Stanley B, editors. Handbook of research ethics in psychological science; 2021. American Psychological Association. p. 83–97.

5. Regev A, Teichmann S, Rozenblatt-Rosen O, Stubbington M, Ardlie K, Amit I et al. The Human Cell Atlas White Paper. 2018.

6. Ball MP, Bobe JR, Chou MF, Clegg T, Estep PW, Lunshof JE et al. Harvard Personal Genome Project: lessons from participatory public research. Genome Medicine 2014; 6:10.

7. Sneddon TP, Li P, Edmunds SC. GigaDB: announcing the GigaScience database. GigaScience 2012; 1:1.

8. Abrams, M. B., Bjaalie, J. G., Das, S., Egan, G. F., Ghosh, S. S., Goscinski, W. J., ... & Martone, M. E. (2022). A standards organization for open and FAIR neuroscience: the international neuroinformatics coordinating facility. Neuroinformatics, 20(1), 25-36.

9. Martone, M. E. (2022). A decade of GigaScience: the importance of community organizations for open and FAIR efforts in neuroinformatics. GigaScience, 11, giac060.

10. Rahimzadeh, V., Dyke, S. O., & Knoppers, B. M. (2016). An international framework for data sharing: moving forward with the global alliance for genomics and health. Biopreservation and biobanking, 14(3), 256-259.

11. Knoppers, B. M. (2014). International ethics harmonization and the global alliance for genomics and health. Genome Medicine, 6(2), 1-3.

12. Valdes- Sosa, P. A., Leon, M. A. B., Lopera, F., Li, M., Bosch- Bayard, J., Bringas, M. L., & Valdes, M. (2022). EEG in the Global Brain Consortium, aiming to strengthen linkages between neuroscientists across borders and disciplines to advance equitable solutions to priority health challenges worldwide. Alzheimer's & Dementia, 18, e059945.

13. Halchenko Y, Meyer K, Poldrack B, Solanky D, Wagner A, Gors J et al. DataLad: Distributed System for Joint Management of Code, Data, and Their Relationship. Journal of Open Source Software 2021 6:63, 3262.

14. Sharif T, Rioux P, Rousseau M, Kassis N, Beck N, Adalat R, et al. CBRAIN: A Web-Based, Distributed Computing Platform for Collaborative Neuroimaging Research. Frontiers in Neuroinformatics 2014; 8:54.

15. Beauvais MJS, Illes J, Knoppers BM. A Marathon, Not a Sprint - Neuroimaging, Open Science and Ethics. Neuroimage. 2021; 1:236:118041.

16. Thorogood A. Policy-Aware Data Lakes: A Flexible Approach to Achieve Legal Interoperability for Global Research Collaborations. Journal of Law and the Biosciences, 2020; 7:1.

17. Bernier A, Knoppers BM. Longitudinal Health Studies: Secondary Uses Serving the Future. Biopreservation and Biobanking. 2021; 19:5 404-413

18. Luo J, Wu M, Gopukumar D & Zhao Y. Big data application in biomedical research and health care: a literature review. Biomedical informatics insights. 2016; 8:BII-S31559.

19. Navale V, Bourne PE. Cloud Computing Applications for Biomedical Science: A Perspective. PLoS Comput Biol 208; 14:6: e1006144.

20. Cirillo D, Valencia A. Big data analytics for personalized medicine. Current opinion in biotechnology. 2019; 58:161-167.

21. Boers SN, van Delden JJN, Bredenoord AL. Broad Consent is Consent for Governance. The American Journal of Bioethics. 2015; 15:9 53-55.

22. Devriendt T, Borry P, Shabani M. Factors that influence data sharing through data sharing platforms: A qualitative study on the views and experiences of cohort holders and platform developers. *PLOS One* 2021; 16:7: e0254202.

23. Corpas M, Kovalevskaya NV, McMurray A, Nielsen FG. A FAIR Guide for Data Providers to Maximise Sharing of Human Genomic Data. PLoS Comput Biol 2018; 14:3: e1005873.

24. Shabani M, Borry P. "You want the right amount of oversight": interviews with data access committee members and experts on genomic data access. Genet Med 2016; 18: 892–897.

25. Cavoukian C et al. CONP Ethics and Data Governance Framework. Canadian Open Neuroscience Platform, 2019.

26. Mittelstadt, B. (2017). From individual to group privacy in big data analytics. Philosophy & Technology, 30(4), 475-494.

27. Bloustein, E. J. (1976). Group privacy: The right to huddle. Rutgers-Cam LJ, 8, 219.

28. Wallace SE, Kirby E, Knoppers BM. How can we not Waste Legacy Genomic Data? Front Genet. 2020; 11:446.

29. Global Alliance for Genomics and Health. Consent Clauses for Genomic Research. Global Alliance for Genomics and Health Regulatory and Ethics Toolkit. 2020.

30. Knoppers BM, Beauvais MJS, Cavoukian A, Clarkson J, Green-Noble L, Illes J et al. Canadian Open Neuroscience Platform (CONP) Ethics Toolkit. 2022.

31. Bernier A, Molnár-Gábor F, Knoppers BM. The International Data Governance Landscape. Journal of Law and the Biosciences 2022; 9:1 :lsac005.

32. Sansone SA, Gonzalez-Beltran A, Rocca-Serra P, Alter G, Grethe JS, Xu H et al. DATS, the data tag suite to enable discoverability of datasets. Sci Data 2017; 4:170059.

.

Figure

Click here to access/download;Figure;Figure 1.png

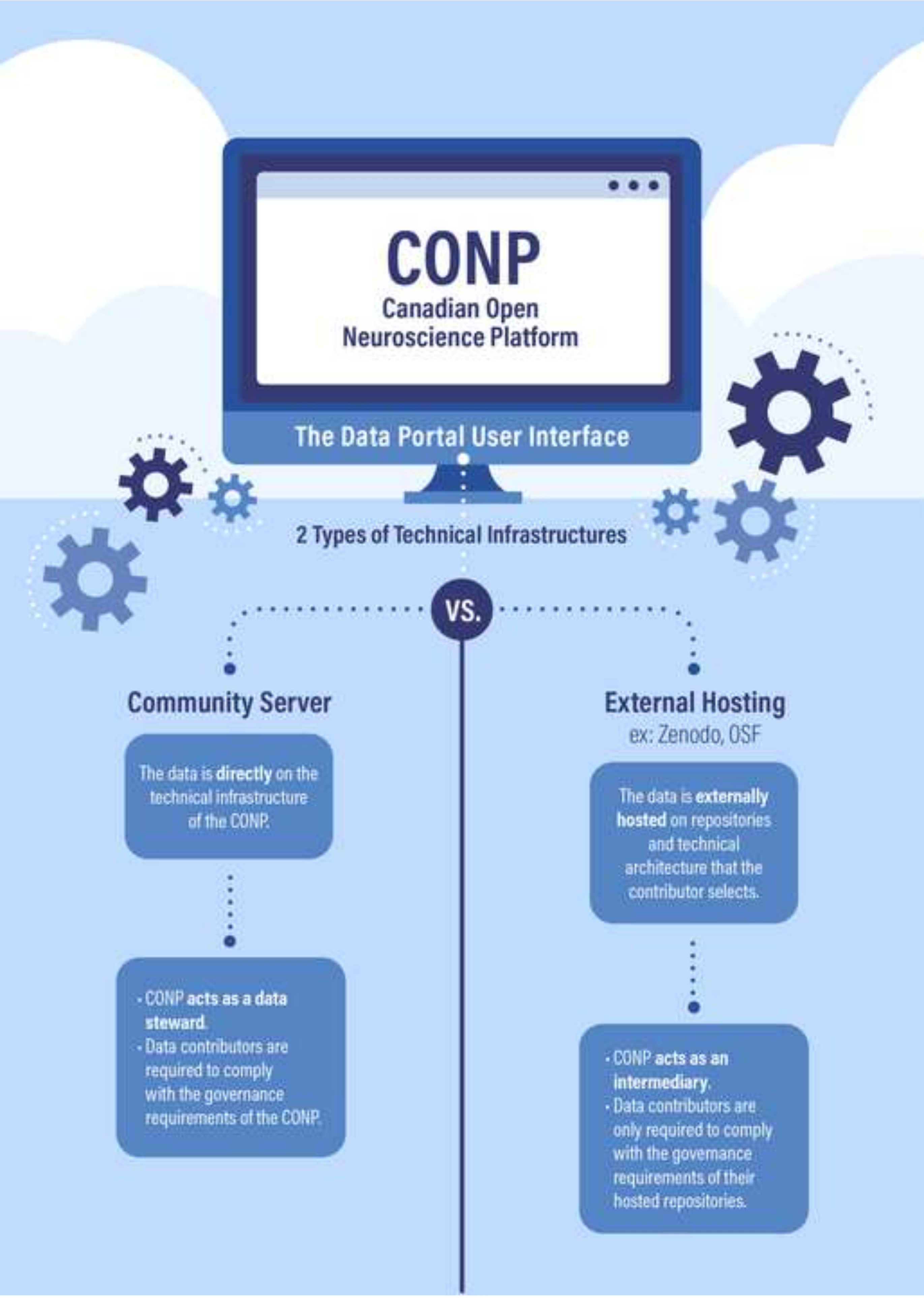

Click here to access/download
Supplementary Material
final manuscript file - changes tracked.docx
