# Supplementary material for: Open Data Governance at the Canadian Open Neuroscience Platform (CONP): From the Walled Garden to the Arboretum
Source: Gigascience. 2024 Jan 13;13:giad114. doi: 10.1093/gigascience/giad114 (PMC10787360; doi:10.1093/gigascience/giad114)
Supplement: giad114_Response_to_Reviewer_Comments_Original_Submission [file giad114_response_to_reviewer_comments_original_submission.pdf]

Author's Response To Reviewer Comments

Response to peer reviewers for the manuscript entitled “Open Data Governance at the Canadian Open Neuroscience Platform (CONP): From the Walled Garden to the Arboretum” (manuscript number: GIGA-D-23-00204)

We sincerely thank the reviewers for their thoughtful and in-depth contributions to the development of this manuscript.

Reviewer 1:

Comment:
However, I will absolutely under no circumstances accept this point made by the authors about how metadata may be kept

"...and the GitHub open software repository to host the dataset metadata"

The authors seem to believe erroneously that GitHub is an open software repository that can permanently host dataset metadata. While this may be relatively true for pair repositories this is not generally true. They fail to understand that this open software repository is not persistent. They may not recommend ANYWHERE (manuscript or on their website) the use of GitHub as a repository! GitHub is owned by Microsoft and removes or eliminates repositories that are no longer actively developed much to the dismay of many grad students and postdocs who have had to beg Microsoft to bring back their data, with little success. The only way to guarantee anything from GitHub is to push a copy of the repo to Zenodo. Unless the policy at GitHub has recently changed, which I would need evidence for, this point must be clearly addressed.

Response:
We understand and are sensitive to the concern the reviewer raises here. In addressing it, we first offer the following points of clarification:
1) Our text makes no claims or suggestions as to the permanence of data hosted on GitHub and, indeed, it would be silly to claim the permanence of any store, whether commercial or institutional. Like any repository, we have policies and practices for best-effort preservation which we adhere to seriously. As is the case for identifiers such as DOIs and the objects they point to, we should rather speak of persistence and not permanence. In our examination (which we believe is consistent with that of the wider community), pragmatically speaking, GitHub is not meaningfully less persistent a resource than others. We did not undertake this design choice lightly or out of expedience but with full consideration of the role GitHub has come to occupy in the research community, though we agree there remain live issues concerning how the community as a whole uses GitHub.
2) The CONP Portal does not rely exclusively on GitHub to store dataset metadata. Indeed, the copy of each dataset’s metadata (in the form of a “DATS.json” file) that is available on the Portal is hosted on the CONP’s local servers and not GitHub.
3) Though we were unable to find a stated policy of removing inactive repositories, and research into community experiences only revealed deletion under unusual circumstances (e.g., serious violation of policy or loss of access to original repo), we are concerned by the reviewer’s experience with GitHub and thank them for this relevant warning. We will further investigate the conditions under which GitHub manages low-activity repositories, the rare conditions under which a deletion might occur, and the warning provided to users in such cases.
4) Though GitHub does not seem any less reliable than typical institutional stores, we again emphasize that the exceedingly unlikely deletion of a dataset’s GitHub-hosted metadata would require intervention from the CONP’s staff to restore updatability and version tracking but it would not affect the availability of the dataset or its metadata to the community as offered through the CONP Portal.

A footnote has been added to the manuscript as a point of clarification:

"Metadata are also stored locally on the CONP Portal's servers and accompany every dataset, whether through browser-based access or through DataLad."

Comment:
A major omission of this work is also in the lack of discussion of the role of INCF in policy, standards setting and the contribution of this important international organization in this space. As far as I understand it, the CONP took significant work from INCF and enhanced it, but this manuscript does not address the role that INCF has played.

Response:
The CONP Principal Investigator and the CONP personnel responsible for the ethics and governance portfolio of the platform throughout its lifetime have been consulted to address this issue. Based on these conversations, our understanding of the relationship between the INCF and the CONP is the following.

The INCF has been engaged in significant standard-development efforts in a number of areas related to neuroinfomatics, which, indeed, is among the reasons the CONP, from its very inception, established a fruitful partnership and collaboration with the INCF. Having reviewed the history of the development of the CONP's Ethics & Governance policies presented in this manuscript with the personnel responsible for its elaboration, to the knowledge of the authors, the CONP has not directly used prior technical or governance approaches that the INCF developed as the basis of its own technologies and practices. Many of the CONP's approaches are founded chiefly on the work of Global Alliance for Genomics and Health.

Needless to say, the CONP does not operate in a vacuum and it was certainly not the intent of the authors to passively diminish the importance of any contributors to this space, one to which the CONP itself wishes to contribute. Some text has been added to acknowledge the INCF's contributions alongside a few other entities. The relevant sections now reads:

"The Canadian Open Neuroscience Platform (CONP) is among an increasing number of international initiatives working to develop policy standards for the open and unrestricted sharing of human biomedical research data. Other examples of organizations who have fostered the development of policies, standards, and tools that facilitate the interoperable sharing of neuroscience data include the Personal Genome Project, the GigaScience GigaDB, the Human Cell Atlas, the Global Alliance for Genomics and Health (GA4GH), and the International Neuroinformatics Coordinating Facility (INCF) [5, 6, 7, 8, 9, 10, 11, 12]."

Comment:
On a more minor point, it would be useful if CONP would create some sort of ontology based search or at least would use a standard lemenalization library in their search box. I searched for Alzheimers and got 0 results while Alzheimer brought back several studies. That omission of simple search technologies is simply unacceptable in the age of google, and Chat bots.

Response:
We very much agree that the CONP Portal's search functionality needs improvement and feature extension, including ontology-based search as the reviewer suggests. Such features are among the Portal's technical roadmap items we hope to implement over the next year of development work.

Reviewer 2:

Comment:
My only concern is the manner in which CONP handles complex cases.

Some countries may have restrictions on using data outside of a clinic for the purpose of sharing, while others may have no specific legal regulation on data ownership, albeit with administrative rules that would lead to a situation in which there are no declared boundaries or rules to be followed when sharing data. In nations where the majority of clinical data are collected in state-owned hospitals, data sharing may be difficult. Does CONP facilitate a mechanism that not only enables the sharing of current data but also

encourages the sharing of potential data in an effort to increase the volume of such data? This may increase the need for a committee to supervise the platform, evaluate data submissions, and, most importantly, provide direction on data sharing procedures. In addition, since there are already clear and straightforward procedures (such as self-checklists), difficult cases requiring assistance from more specialists would be eliminated from the start. In other words, some researchers would be irritated by the clear-cut standard and somewhat automated processing of CONP. (such as self-checklist), which would eliminate difficult cases that require the assistance of more specialists.

Response:
These considerations are indeed quite valid. The design of the CONP governance approach is not intended to displace or replace the more fulsome approaches to governance that other platforms have integrated. Rather – it is intentionally designed to foster pluralism in the governance and stewardship conditions that can be applied to data through the platform. To this end, datasets that require more specialised approaches to governance can be either uploaded to other repositories that have implemented the required stewardship measures and can be made discoverable and accessible through the CONP Portal or (in specific cases that continue to broaden with the Portal's technical development) through credentialed access to data stored in a specific data-management system (e.g., the LORIS platform) which nonetheless allow access through the Portal. These are currently available options for datasets that require more thorough governance and access control.

Further to this, the CONP regularly engages in open dialog with potential data contributors to help evaluate and plan both retrospective and prospective data contributions that account for the governance obligations of both parties. The latter case often involves advice concerning the elements of informed consent and data anonymization the CONP recommends generally and, in the case where CONP servers are used to host data, are required. In this way, we hope to encourage broader open-science practice through some consideration of the possibility of open data-sharing as early as possible in the planning stages of a study. Doing so greatly facilitates matters down the line. The types of interactions with the community feed back into the ongoing work of CONP's Ethics & Governance committee and its endeavour to respond to questions regarding data deposit or governance. In some cases, these efforts may take the form of iterative dialogues that result in bespoke safeguards or paths to data deposit for specific communities.

To clarify these nuances in the text, we have added certain elements to the manuscript (changes are highlighted in bold):

"In addition to performing the foregoing functions, the Ethics and Data Governance Committee provides ad-hoc guidance to the operational staff of the CONP and to researchers who intend to deposit data on the CONP Portal, responding to governance challenges as these arise. This includes tailoring the CONP guidance tools to respond to new risks or requirements and providing counsel on their application. The Ethics and Data Governance Committee further monitors for potential risks associated to the upload of specific categories of data, specific populations, or circumstances of data upload that might require the imposition of additional governance controls on a contextual basis, especially where communities indicate that data pertaining to their members requires additional governance safeguards to mitigate the potential for public data dissemination to lead to group-level harms (15, 26, 27)."

Reviewer 3:

Comments (not replicated here in full – but have addressed them in the comments below):

Response:
The considerations raised throughout this peer review are deeply perceptive, forward-looking, and rich with detail. The prospective alignment of consent practices and de-identification processes to those of the repositories in which data are anticipated to be deposited is a common precondition to data contribution. Repositories define their consent and identifiability requirements as a matter of habitual course.

The heightened emphasis that the manuscript places on the foregoing governance safeguards is not intended to suggest that individual-facing safeguards such as data de-identification and consent are the

sole mechanisms that should be implemented to safeguard research participants against harms, or that group harms should not be safeguarded against. Defining consent and de-identification practices is accorded great importance in bioethics literature generally, and especially in the binding Research Ethics requirements detailed in the TCPS-2. One of the principal aims of this manuscript is to detail how repositories can interface with the consent and de-identification requirements that are applicable to researchers in depositing data in such repositories, so as to help researchers prepare their data for the release of data in full open-access and determine when this is appropriate. For this reason, a great deal of the word-count has been dedicated to addressing these issues.

The mitigation of group harms – and addressing population-specific concerns – often occurs through the engagement of the Research Ethics Committees (RECs) in the oversight processes that are dedicated to individual studies. Because this occurs prior to the deposit of data in repositories, it is not addressed at the stage of administering the contribution of data to repositories.

The manuscript is directed to establishing practicable approaches to sharing data that have been consented and de-identified in a manner that renders its open release lawful and compliant with research ethics guidance. Much of the test is therefore used to explore and outline the approaches that the CONP has at present adopted to enable oversight – at the level of the repository – of data which is intended for sharing in full open access.

Finding practical mechanisms to manage and to address potential group harms or population-specific risks and data governance practices is nonetheless an extremely important endeavor, one in which the CONP is also engaged.

Text has been added to this manuscript stating that the CONP Ethics and Data Governance Committee is devising specialised governance controls for specific populations that act as additional safeguards to deposit their data in full open access, and emphasizes the importance thereof to making open science more inclusive and equitable:

The first section of this text elaborates the important role of Research Ethics Committees and other stakeholders in addressing these categories of concerns:

“The CONP governance model responds to prevailing legal paradigms and applicable ethics requirements, to enable the open sharing of neuroscience data consented or permissioned for open release. The mitigation of population-specific or group-specific harms is intermediated through the involvement of Research Ethics Committees (RECs) that oversee research or through other stakeholders, such as patient communities and population-specific research organizations, prior to the upload of individuals’ coded data to the CONP. These actors mitigate such risks in overseeing the drafting of informed consent materials, determining how data can be collected, de-identified, and released, and assessing whether a particular data repository is suitable for the deposit of data. In the future, collaboration with stakeholders from relevant populations will be required to tailor the governance approach of the CONP to the risks that affect specific vulnerable groups, communities of patients with unique needs, and indigenous communities. This could require additional measures to tailor consent procedures to account for population-specific risks, for example to enable data contribution from those persons that do not have legal capacity to provide informed consent on their own behalf (e.g., pediatric populations, or patients that suffer from neurodegenerative diseases), or to implement population-specific de-identification or data manipulation processes that mitigate relevant group-level harms. This will further enable the dissemination of data relative to populations underrepresented in research datasets through the CONP.”

As noted in the response to one of Reviewer 2’s comments, text has been added to highlight that the CONP Ethics and Data Governance Committee further considers the implementation of population-specific or group-specific safeguards on an ongoing basis (as highlighted):

“In addition to performing the foregoing functions, the Ethics and Data Governance Committee provides ad-hoc guidance to the operational staff of the CONP and to researchers that intend to deposit data on the CONP, responding to governance challenges as these arise. This includes tailoring the CONP guidance tools to respond to new risks or requirements and providing counsel on their application to specific factual

circumstances. The Ethics and Data Governance Committee further monitors for potential risks associated to the upload of specific categories of data, specific populations, or circumstances of data upload that might require the imposition of additional governance controls on a contextual basis, especially where communities indicate that data pertaining to their members requires additional governance safeguards to mitigate the potential for public data dissemination to lead to group-level harms (15; 26; 27).”

In addition to the incorporation of this text – the CONP is experimenting with a number of additional safeguards at this time. These are not listed in the manuscript as their design and implementation are still in their early phases.

One further note is that Figure 1 has been added to manuscript to further improve the clarity of its presentation of the governance models available to researchers that deposit data in the CONP.
