# Supplementary material for: Open Data Governance at the Canadian Open Neuroscience Platform (CONP): From the Walled Garden to the Arboretum
Source: Gigascience. 2024 Jan 13;13:giad114. doi: 10.1093/gigascience/giad114 (PMC10787360; doi:10.1093/gigascience/giad114)
Supplement: giad114_Response_to_Reviewer_Comments_Revision_1 [file giad114_response_to_reviewer_comments_revision_1.pdf]

Author's Response To Reviewer Comments

Response to the peer reviewers

To whom this may concern,

We wish to offer our most sincere thanks for the thoughtful insights which you have provided on our manuscript.
We have edited the manuscript according to your recommendations.

Comment:
1. I would like authors to strengthen the wording in their new footnote, something such as "because GitHub has no persistence policy to maintain no longer updated repositories, CONP maintains …" since people do not believe this until they have either seen it a bunch or until their repo is removed, this needs to be explicit.
….also acceptable would be a direct link to the persistence policy from GitHub for how they handle code and data repositories, especially inactive repositories. GitHub's definition of inactive would be useful to discuss if authors choose this route.

Response:
As we stated in our previous response, we too are worried about what the reviewer describes as their experience with GitHub, especially as the research community has become so heavily dependent on GitHub as the de facto code repository for research. As with most organizations, their 'persistence policy' is stated in the negative, that is, they state the conditions under which content can be removed:
https://docs.github.com/en/site-policy/content-removal-policies
These are quite conventional and do not include repository inactivity. We also found a "GitHub Deceased User Policy" and a "GitHub Government Takedown Policy":
https://docs.github.com/en/site-policy/other-site-policies/github-deceased-user-policy
https://docs.github.com/en/site-policy/other-site-policies/github-government-takedown-policy
These too are quite conventional and so, while not doubting the reviewer's experience, we remain at pains to explain it.
Given GitHub's "Content Removal Policies" cited above and the impermanent nature of these policies in general, we feel it would be neither accurate to state GitHub has no persistence policy nor wise to rely on whatever their current policy is (even it positively affirmed what we would prefer to see). We therefore propose that the footnote read as follows:
Persistence policies for repositories such as GitHub can change at any moment. For this and other reasons, such as facilitating provenance tracking, metadata are also stored locally on the CONP Portal's servers and accompany every dataset, whether through browser-based or DataLad access.

Comment:
2. Authors state that they have included RRIDs in their manuscript, and they have not. Why mislead reviewers?

Response:
It was not our intention to suggest that the manuscript contained RRIDs. We have contacted the journal editors in this respect. They have confirmed that no RRIDs are required in the submission of a review article.
The notice that an RRID was included is an automated inclusion made by the submission portal. We stand to clarify that the manuscript does not contain an RRID. No notice to this effect will be comprised in the finalized manuscript.

***
In addition to these changes, we have made some minor grammar edits, standardized our use of the word data throughout the entire manuscript (i.e., data are rather than data is), and tweaked the figure slightly for accuracy.

Sincerest thanks to both the reviewers and the editors for their prescient, thorough, and efficient contributions to the manuscript. The final manuscript is much strengthened as a result.

With warm wishes
The authors
