# Supplementary material for: Open Data Governance at the Canadian Open Neuroscience Platform (CONP): From the Walled Garden to the Arboretum
Source: Gigascience. 2024 Jan 13;13:giad114. doi: 10.1093/gigascience/giad114 (PMC10787360; doi:10.1093/gigascience/giad114)
Supplement: giad114_Reviewer_1_Report_Original_Submission — Anita Bandrowski -- 8/11/2023 Reviewed [file giad114_reviewer_1_report_original_submission.pdf]

Reviewer Report

Title: Open Data Governance at the Canadian Open Neuroscience Platform (CONP): From the Walled Garden to the Arboretum

Version: Original Submission **Date:** 8/11/2023

Reviewer name: Anita Bandrowski

Reviewer Comments to Author:

I have reviewed the manuscript entitled "Open Data Governance at the Canadian Open Neuroscience Platform (CONP): From the Walled Garden to the Arboretum"
This manuscript is clearly written and will be valuable to the community as a means to explain what the CONP is and what is its unique contribution.
However I will absolutely under no circumstances accept this point made by the authors about how metadata may be kept
"...and the GitHub open software repository to host the dataset metadata"
The authors seem to believe erroneously that GitHub is an open software repository that can permanently host dataset metadata. While this may be relatively true for pair repositories this is not generally true. They fail to understand that this open software repository is not persistent. They may not recommend ANYWHERE (manuscript or on their website) the use of GitHub as a repository! GitHub is owned by Microsoft and removes or eliminates repositories that are no longer actively developed much to the dismay of many grad students and postdocs who have had to beg Microsoft to bring back their data, with little success. The only way to guarantee anything from GitHub is to push a copy of the repo to Zenodo. Unless the policy at GitHub has recently changed, which I would need evidence for, this point must be clearly addressed.
A major omission of this work is also in the lack of discussion of the role of INCF in policy, standards setting and the contribution of this important international organization in this space. As far as I understand it, the CONP took significant work from INCF and enhanced it, but this manuscript does not address the role that INCF has played.
On a more minor point, it would be useful if CONP would create some sort of ontology based search or at least would use a standard lemenalization library in their search box. I searched for Alzheimers and got 0 results while Alzheimer brought back several studies. That omission of simple search technologies is simply unacceptable in the age of google, and Chat bots.

Methods

Are the methods appropriate to the aims of the study, are they well described, and are necessary controls included? Choose an item.

Conclusions

Are the conclusions adequately supported by the data shown? Choose an item.

Reporting Standards

Does the manuscript adhere to the journal's guidelines on minimum standards of reporting? Choose an item.

Choose an item.

Statistics

Are you able to assess all statistics in the manuscript, including the appropriateness of statistical tests used? Choose an item.

Quality of Written English

Please indicate the quality of language in the manuscript: Choose an item.

Declaration of Competing Interests

Please complete a declaration of competing interests, considering the following questions:

- Have you in the past five years received reimbursements, fees, funding, or salary from an organisation that may in any way gain or lose financially from the publication of this manuscript, either now or in the future?
- Do you hold any stocks or shares in an organisation that may in any way gain or lose financially from the publication of this manuscript, either now or in the future?
- Do you hold or are you currently applying for any patents relating to the content of the manuscript?
- Have you received reimbursements, fees, funding, or salary from an organization that holds or has applied for patents relating to the content of the manuscript?
- Do you have any other financial competing interests?
- Do you have any non-financial competing interests in relation to this paper?

If you can answer no to all of the above, write 'I declare that I have no competing interests' below. If your reply is yes to any, please give details below.

none

I agree to the open peer review policy of the journal. I understand that my name will be included on my report to the authors and, if the manuscript is accepted for publication, my named report including any attachments I upload will be posted on the website along with the authors' responses. I agree for my report to be made available under an Open Access Creative Commons CC-BY license (http://creativecommons.org/licenses/by/4.0/). I understand that any comments which I do not wish to be included in my named report can be included as confidential comments to the editors, which will not be published.

Choose an item.

To further support our reviewers, we have joined with Publons, where you can gain additional credit to further highlight your hard work (see: https://publons.com/journal/530/gigascience). On publication of this paper, your review will be automatically added to Publons, you can then choose whether or not to claim your Publons credit. I understand this statement.

Yes Choose an item.
